# Are infections associated with cognitive decline and neuroimaging outcomes? A historical cohort study using data from the UK Biobank study linked to electronic health records

**DOI:** 10.1038/s41398-022-02145-z

**Published:** 2022-09-15

**Authors:** Rutendo Muzambi, Krishnan Bhaskaran, Christopher T. Rentsch, Liam Smeeth, Carol Brayne, Victoria Garfield, Dylan M. Williams, Nish Chaturvedi, Charlotte Warren-Gash

**Affiliations:** 1 Faculty of Epidemiology and Population Health, London School of Hygiene and Tropical Medicine, London, WC1E 7HT UK; 2 Section of General Internal Medicine, Yale School of Medicine, New Haven, CT 06510 USA; 3 Cambridge Institute of Public Health, Cambridge University, Cambridge, UK; 4 MRC Unit for Lifelong Health and Ageing at UCL, Institute of Cardiovascular Science, University College London, 1-19 Torrington Place, London, WC1E 7HB UK; 5 Department of Medical Epidemiology and Biostatistics, Karolinska Institutet, Stockholm, Sweden

**Keywords:** Diseases, Pathogenesis

## Abstract

While there is growing evidence of associations between infections and dementia risk, associations with cognitive impairment and potential structural correlates of cognitive decline remain underexplored. Here we aimed to investigate the presence and nature of any associations between common infections, cognitive decline and neuroimaging parameters. The UK Biobank is a large volunteer cohort (over 500,000 participants recruited aged 40–69) with linkage to primary and secondary care records. Using linear mixed effects models, we compared participants with and without a history of infections for changes in cognitive function during follow-up. Linear regression models were used to investigate the association of infections with hippocampal and white matter hyperintensity (WMH) volume. 16,728 participants (median age 56.0 years [IQR 50.0–61.0]; 51.3% women) had baseline and follow-up cognitive measures. We found no evidence of an association between the presence of infection diagnoses and cognitive decline for mean correct response time (slope difference [infections versus no infections] = 0.40 ms, 95% CI: −0.17–0.96 per year), visual memory (slope difference 0.0004 log errors per year, 95% CI: −0.003–0.004, fluid intelligence (slope difference 0.007, 95% CI: −0.010–0.023) and prospective memory (OR 0.88, 95% CI: 0.68–1.14). No evidence of an association was found between infection site, setting or frequency and cognitive decline except for small associations on the visual memory test. We found no association between infections and hippocampal or WMH volume. Limitations of our study include selection bias, potential practice effects and the relatively young age of our cohort. Our findings do not support a major role for common midlife infections in contributing to cognitive decline for this cohort. Further research is warranted in individuals with more severe infections, for infections occurring later in life.

## Introduction

Growing evidence from longitudinal studies supports the role of common infections such as sepsis [1–4], pneumonia [4, 5], other lower respiratory tract infections (LRTIs) [4], urinary tract infections (UTIs) [4, 5], and skin and soft tissue infections (SSTIs) [4, 5], in increasing the risk of dementia, though some findings have been conflicting [6].

Dementia has a long preclinical phase which can take decades to develop [7]. Before the clinical expression of dementia, cognitive and neuropathological changes associated with dementia progression can be observed but it is unclear whether infections are relevant during this process [8, 9]. Identifying the point at which infections may act before clinical onset of dementia might allow interventions to be targeted and timed appropriately to prevent or delay the onset of dementia.

Infection-related hospitalisations, particularly for sepsis and pneumonia, have been associated with cognitive decline [10–14]. However, there is limited understanding of the relationship of other sites of common infections diagnosed in different clinical settings such as primary care with changes in cognitive function over time. Other limitations of existing studies include either relatively small study sizes, the use of a single global measure of cognition rather than individual cognitive domains, or inadequate confounder adjustment, given the wide range of potential confounders [15–24].

A possible link between infections and other subclinical markers of dementia risk such as hippocampal atrophy and white matter hyperintensities (WMH) may exist. However, evidence on the link between common infections and these neuroimaging markers is scarce. Compared to cognitive function measures, neuroimaging measures may be less prone to the effects of sociodemographic influences such as education; investigating both cognitive decline and neuroimaging measures may allow us to triangulate our findings across different outcomes associated with dementia risk.

Therefore, we aimed to explore the association between common infections and subclinical markers of dementia (cognitive decline, hippocampal volume and WMH volume) in a population without pre-existing dementia or evidence of cognitive impairment. We then assessed whether these associations differed by infection site, clinical setting, frequency and timing of infections.

## Methods

### Study design and population

We used data from the UK Biobank study, an ongoing prospective study which recruited over 500,000 participants aged 40–69 between 2006 and 2010 from 22 assessment centres based in England, Wales and Scotland [25]. Over 9 million individuals registered with the National Health Service (NHS) who lived within 40 km of one of the UK Biobank assessment centres were invited to take part in the study via postal invitations, however, the response rate was low with 5.5% consenting to take part in the study and attending the baseline assessment [25, 26]. The methodology of the UK Biobank has been described previously and is summarised in the Supplementary Information [27].

**Fig. 1:**
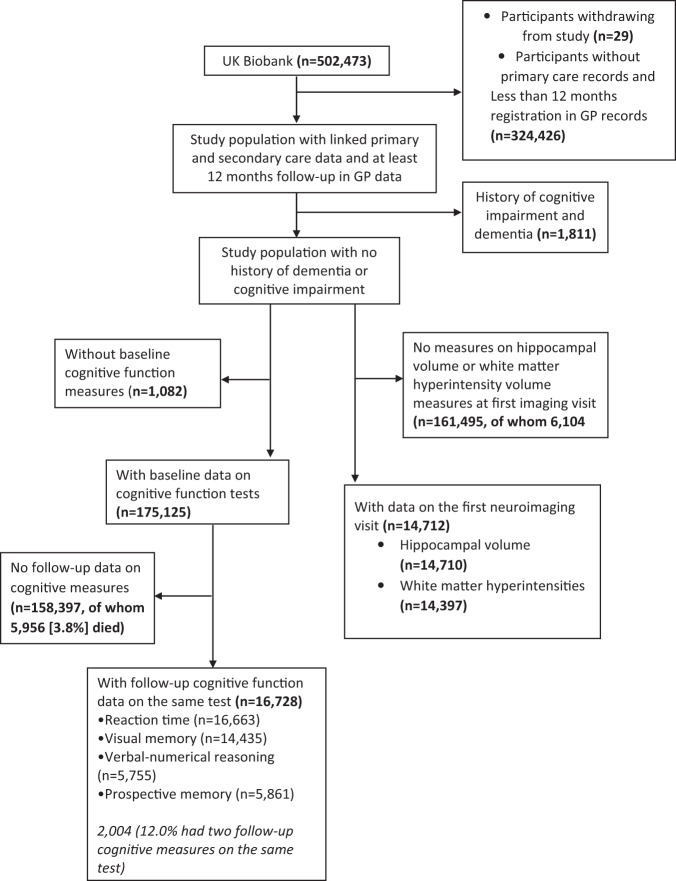
Flow chart of study population. Flowchart depicting participants included and excluded from this study.

Linkage to primary care data for ~45% of the cohort has been made available by UK Biobank for non-COVID research [28]. To minimise exposure misclassification, our study population was limited to only participants with linked primary and secondary care records (Fig. 1). We excluded participants who had less than 12 months registration with a GP practice to avoid incorporating historical diagnoses when defining our exposure as previous studies suggest historical diagnoses may be recorded within the first 12 months when a patient registers with a GP practice [29]. We defined baseline as the date participants attended the UK Biobank baseline assessment. A subset of participants was invited to attend the first repeat assessment (2012–13) and the first wave of the neuroimaging assessments (2014 onwards). Cognitive function was assessed at all three assessments.

Our study explored two subsets of the UK Biobank population, a cognition subset, and a separate neuroimaging subset. For the cognition subset, we included participants with valid measures of cognitive function completed at baseline and at least one follow-up measure on the same test. For our neuroimaging cohort, we included only participants with data on hippocampal or WMH volume who attended the first neuroimaging visit. We excluded participants with dementia or cognitive impairment at baseline in both cohorts using self-report data and linked primary and secondary care records. We excluded participants with a history of dementia or cognitive impairment because the aim of our study was to investigate whether infections were associated with earlier or subclinical markers of dementia risk thus we were specifically interested in the stage before clinical expression of dementia.

### Exposures

Our exposures were one or more of the following common infections: sepsis, pneumonia, other LRTIs, UTIs and SSTIs. We examined these as exposures in combination and separately. Sepsis is defined as a serious, life-threatening condition caused by a dysregulated host response following a range of infections [30]. Infections were identified in the 5 years up to UK Biobank participants’ baseline assessment visit. This time period of 5 years was chosen due to issues with the completeness of historical linked primary care data. Infections were defined using Read codes in linked primary care records and International Classification of Diseases (ICD)-10 codes in hospital records. To increase the specificity of our infection definition, participants were defined as having UTIs or SSTIs if they were also prescribed antibiotics on the same date as infection diagnosis.

### Outcomes

#### Cognitive function

Participants completed a 15 min battery of computerised cognitive function tests including measurement of reaction time which is referred to in this study as the mean correct response time test, visual memory (pairs matching test), fluid intelligence (verbal-numerical reasoning) and prospective memory. Details of these tests and their association with age have been reported elsewhere [31, 32]. Our outcome measures for these tests were: (1) mean correct response time (milliseconds) taken for a participant to correctly identify matching pairs of cards (Data-field 404), (2) total number of incorrect matches in participants who completed the visual memory test (Data-field 399), (3) total number of incorrect answers on the fluid intelligence test (Data-field 20016) and (4) participants were scored 0 for the correct answer and 1 for an incorrect answer at the first attempt for the prospective memory test (one minus Data-field 20018). Lower scores in all cognitive tests indicated better performance. We dealt with outliers for mean correct response time using an approach consistent with the method outlined by the UK Biobank. We therefore excluded response times under 50 milliseconds due to anticipation and response times over 2000 milliseconds were excluded as the UK Biobank indicates that the cards used for this web-based test had disappeared by then.

#### Neuroimaging measures

We used measures of hippocampal volume (Data-fields 25019 and 25020) with lower volumes being indicative of Alzheimer’s disease pathology and WMH volume (Data-field 25781) with higher volumes indicative of cerebral small vessel disease pathology measures from the first imaging visit (Supplementary Information). We assessed the total volume of WMH volume using postprocessed measures from T1 and T2 weighted fluid attenuation inversion recovery (FLAIR) imaging technique derived by the UK Biobank study [33, 34].

#### Covariates

Data from baseline assessment questionnaires, verbal interview and linked primary and secondary care data were used to define covariates. Demographic variables included age (years), sex, ethnicity (White European, South Asian, African or Caribbean, Mixed or Other) and years in full-time education based on the International Standard Classification of Education (ISCED) 1997 (Supplementary Table 1) [35, 36]. Socioeconomic status was measured using the Townsend Deprivation scores [37]. Potential lifestyle factors included body mass index (BMI, kg/m^2^), smoking, alcohol intake frequency and physical activity. Diabetes was ascertained using HbA1c and all data sources mentioned above. Other comorbidities included anxiety and depression, asthma, chronic kidney disease, chronic liver disease, chronic obstructive pulmonary disease, heart failure, hypertension, inflammatory bowel disease, multiple sclerosis, obstructive sleep apnoea, rheumatoid arthritis, psoriasis, severe mental illness, stroke and traumatic brain injury (Supplementary Information). Code lists for covariates ascertained in electronic health records can be accessed at: 10.17037/DATA.00002573.

Missing data on covariates was minimal (≤3.6% in total) in both cohorts and not all of these covariates were adjusted for in each analysis thus we used a complete case analysis for all analyses (Supplementary Information).

Approval for the UK Biobank study was obtained from the North West Multi-Centre Research Ethics Committee and the present research was conducted under application number 7661. All participants of the study provided written informed consent. Ethical approval was also obtained from the London School of Hygiene and Tropical Medicine research ethics committee (reference number 22721).

### Statistical analysis

We developed a statistical analysis plan before conducting our analyses as shown in the Supplementary Material pages 33–47. We descriptively explored the potential for selection bias in our study by comparing the baseline characteristics of participants included and excluded from our study.

### Association between common infections and cognitive decline

For each continuous cognitive measure (mean correct response time, visual memory and fluid intelligence), we fitted linear mixed models with random intercept and slope effects using an unstructured covariance matrix to estimate the rates of cognitive decline over follow-up in participants with and without a history of infections. Interaction terms were fitted between infections and time since baseline to assess the difference in cognitive decline over follow-up. Due to skewed distribution and zero value inflation of the visual memory error scores, a value of one was added to the scores which were then log_n_-transformed. For the dichotomous test (prospective memory), we used logistic regression models for participants with correct recall at baseline to examine the association between infections and cognitive decline with a binary variable (coded as 0 for correct recall and 1 for incorrect recall at follow-up).

For linear mixed models, minimally adjusted models included age, sex and an interaction term between time and infection. For all other analyses, minimally adjusted models included age and sex. For each analysis, we considered adjustment for all potential confounders described above and adjusted for covariates that changed the main association estimates by an important amount (9% or greater change in association magnitude) in the fully adjusted model.

In our secondary analyses, we investigated the association between site, clinical setting (GP-recorded or hospital-recorded), frequency (with the number of infections modelled as a continuous variable) and timing of infections expressed as year(s) since infection diagnosis in the 5 years up to baseline (0 to <1 year, 1 to <2 years, 2 to <3 years, 3 to <4 years and 4 to 5 years). Given the potential interaction between inflammatory comorbidities, such as diabetes, and dementia pathogenesis, we explored whether the associations of infections on cognition differ by diabetes category (using a binary variable for diabetes) and tested for the presence of effect modification by fitting an interaction term [38]. We performed additional analyses to compare the associations between infections and cognitive decline by age group (40–49, 50–59 and 60+) and sex. This is because increasing age is associated with greater trajectories of cognitive decline and some studies have reported sex differences in cognitive decline [39, 40].

### Association of common infections with hippocampal and WMH volume

We log-transformed WMH volume due to a positively skewed distribution. We used linear regression models to estimate the association between infections and each structural neuroimaging measure. We used the same strategy for confounder adjustment as the approach described for our cognitive decline analyses. To aid interpretation, log-transformed WMH volume was reported using exponentiated betas and was interpreted as percentages. For example, exponentiated beta 1.01 refers to a 1% increase in WMH volume.

### Sensitivity analyses

We conducted a range of sensitivity analyses to test the robustness of our findings. First, we found evidence of non-normal distribution for mean correct response time. However, when we inverse or log transformed this variable, we were unable to fit the models due to unstable standard errors and models failing to converge. Thus, we used raw scores in our main analyses and in a sensitivity analysis we specified an independent covariance structure which allowed us to re-run our models using the inverse transformed variable which showed evidence of a normal distribution (Supplementary Information). Second, we repeated our main analyses excluding participants diagnosed with infections during follow-up to reduce misclassification of our exposure. Third, we repeated our main analyses excluding participants whose current registration date with a GP practice was <5 years. Given that infections were defined within 5 years prior to baseline, this analysis ensured that all participants had at least 5 years of follow-up in which to capture infection diagnoses. Fourth, previous studies suggest infections may have differing associations with the left and right hippocampus thus we repeated our main analyses on hippocampal volume separately for the left and right hippocampus [41].

Statistical analyses were performed in Stata MP (version 16.0) and RStudio (version 4.1.0).

## Results

Of the 176,207 participants with linked primary and secondary care records and no history of cognitive impairment or dementia at baseline, 16,728 (9.5%) had baseline and at least one follow-up cognitive measure on the same test, and of whom 2,004 (12.0%) had two follow-up cognitive measures. 14,712 participants completed neuroimaging measurements at the first imaging visit (Fig. 1). The mean time interval between baseline and the first or second repeat cognitive function assessment was 4.0 years (sd 0.78) and 8.27 years (sd 1.6), respectively. Compared to participants without follow-up cognitive measures, participants included in our study were slightly younger, less likely to be female, had more years in education, fewer infections and performed better on baseline cognitive tests (Table 1). Further descriptive information on infections in participants included and excluded from the study is presented in Supplementary Table 2. 323 (1.0%) participants excluded from the study had infection-related mortality compared to 5 (0.2%) participants included in the study.

**Table 1:** Baseline characteristics of participants included and excluded from the study for the cognitive function and neuroimaging cohorts.

	Cohort with cognitive function measures		Cohort with neuroimaging measures	
Characteristics	Included (*n* = 16,728)	Excluded because of no follow-up cognition measures (158,383)	Included (14,712)	Excluded because of no neuroimaging measures (161,495)
Any Infection	2971 (17.8%)	31,381 (19.8%)	2435 (16.6%)	32,214 (19.9%)
Mean age at baseline assessment (years)	55.59 (7.5)	56.73 (8.1)	54.82 (7.5)	56.79 (8.1)
Median age at baseline assessment (years)	56.0 (50.0–61.0)	58.0 (50.0–63.0)	55.0 (49.0–61.0)	58.0 (50.0–63.0)
Age category (years)				
40–44	1650 (9.9%)	15,744 (9.9%)	1680 (11.4%)	15,824 (9.8%)
45–49	2458 (14.7%)	20,519 (13.0%)	2336 (15.9%)	20,768 (12.9%)
50–54	2887 (17.3%)	23,606 (14.9%)	2781 (18.9%)	23,833 (14.8%)
55–59	3721 (22.2%)	27,994 (17.7%)	3267 (22.2%)	28,645 (17.7%)
60–64	4048 (24.2%)	38,855 (24.5%)	3231 (22.0%)	39,925 (24.7%)
65+	1964 (11.7%)	31,665 (20.0%)	1,417 (9.6%)	32,500 (20.1%)
Women	8576 (51.3%)	87,051 (55.0%)	7781 (52.9%)	88,387 (54.7%)
Ethnicity				
White European	16,323 (97.6%)	150,683 (95.1%)	14,275 (97.0%)	153,339 (94.9%)
South Asian	110 (0.7%)	2776 (1.8%)	135 (0.9%)	2893 (1.8%)
African or Caribbean	76 (0.5%)	1630 (1.0%)	76 (0.5%)	1670 (1.0%)
Mixed or other	177 (1.1%)	2787 (1.8%)	185 (1.3%)	2851 (1.8%)
Missing	42 (0.3%)	507 (0.3%)	41 (0.3%)	742 (0.5%)
Diabetes status				
No diabetes	14,762 (88.2%)	134,585 (85.0%)	13,203 (89.7%)	136,823 (84.7%)
Pre-diabetes	370 (2.2%)	5109 (3.2%)	279 (1.9%)	5256 (3.3%)
Undiagnosed diabetes	1104 (6.6%)	10,157 (6.4%)	884 (6.0%)	10,579 (6.6%)
Controlled diabetes	362 (2.2%)	5593 (3.5%)	256 (1.7%)	5781 (3.6%)
Uncontrolled diabetes	130 (0.8%)	2939 (1.9%)	90 (0.6%)	3056 (1.9%)
Educational attainment (years in full-time education)	16.6 (4.4)	14.7 (5.2)	16.8 (4.3)	14.7 (5.2)
Baseline BMI, kg/m^2^ (mean)	26.7 (4.4)	27.6 (4.8)	26.5 (4.2)	27.6 (4.8)
Townsend deprivation score (mean)	−2.2 (2.5)	−1.4 (3.0)	−2.1 (2.6)	−1.4 (3.0)
Baseline number of days/week moderate physical activity >10 min	3.0 (2.0–5.0)	3.0 (2.0–5.0)	3.0 (2.0–5.0)	3.0 (2.0–5.0)
Smoking status				
Never smoker	12,060 (72.1%)	104,733 (66.1%)	10,670 (72.5%)	106,714 (66.1%)
Ex-smoker	3650 (21.8%)	36,455 (23.0%)	3138 (21.3%)	37,117 (23.0%)
Current smoker	1001 (6.0%)	16,901 (10.7%)	889 (6.0%)	17,141 (10.6%)
Missing	17 (0.1%)	294 (0.2%)	15 (0.1%)	523 (0.3%)
Baseline alcohol intake frequency				
Rarely or never	2,192 (13.1%)	31,244 (19.7%)	1860 (12.6%)	31,965 (19.8%)
1–8 times per month	6159 (36.8%)	59,815 (37.8%)	5,399 (36.7%)	60,854 (37.7%)
16 times per month-every day	8374 (50.1%)	67,161 (42.4%)	7447 (50.6%)	68,290 (42.3%)
Missing	<5	163 (0.1%)	6 (0.0%)	386 (0.2%)
Comorbidities				
Anxiety and depression	1,968 (11.8%)	20,832 (13.2%)	1660 (11.3%)	21,332 (13.2%)
Severe mental illness	187 (1.1%)	2150 (1.4%)	155 (1.1%)	2216 (1.4%)
Inflammatory bowel disease	708 (4.2%)	7788 (4.9%)	611 (4.2%)	7947 (4.9%)
Multiple Sclerosis	51 (0.3%)	612 (0.4%)	41 (0.3%)	630 (0.4%)
Rheumatoid arthritis	155 (0.9%)	2269 (1.4%)	126 (0.9%)	2314 (1.4%)
Psoriasis	460 (2.7%)	4227 (2.7%)	402 (2.7%)	4309 (2.7%)
Asthma	2099 (12.5%)	20,981 (13.2%)	1835 (12.5%)	21,410 (13.3%)
Chronic kidney disease	141 (0.8%)	1950 (1.2%)	119 (0.8%)	1995 (1.2%)
Chronic liver disease	479 (2.9%)	6576 (4.2%)	328 (2.2%)	6789 (4.2%)
Chronic obstructive pulmonary disease	114 (0.7%)	2798 (1.8%)	75 (0.5%)	2881 (1.8%)
Heart failure	208 (1.2%)	4233 (2.7%)	149 (1.0%)	4379 (2.7%)
Hypertension	2613 (15.6%)	33,785 (21.3%)	1982 (13.5%)	34,747 (21.5%)
Obstructive sleep apnoea	126 (0.8%)	1547 (1.0%)	97 (0.7%)	1591 (1.0%)
Stroke	116 (0.7%)	1756 (1.1%)	80 (0.5%)	1815 (1.1%)
Traumatic brain injury	118 (0.7%)	1260 (0.8%)	92 (0.6%)	1299 (0.8%)
Baseline cognitive function test performance				
Mean correct response time score baseline (milliseconds)	540.3 (102.2)	561.3 (118.8)	536.2 (99.8)	549.3 (106.9)
Pairs matching test score (incorrect matches)	5.1 (2.9)	5.50 (3.3)	5.1 (2.8)	5.3 (3.1)
Fluid intelligence test score (incorrect answers)	6.3 (2.0)	7.08 (2.1)	6.25 (2.0)	6.25 (2.0)
Prospective Memory test (incorrect answer)	761 (13.0%)	14,323 (24.6%)	532 (12.7%)	229 (13.7%)

For data protection, table cells containing fewer than 5 participants were recorded as ‘<5’.

Characteristics of participants included in the two subsets of our study, one with follow-up cognitive measures and the other who attended the first imaging visit, are presented in Table 2 and Supplementary Fig. 1. 11, 455 participants were included in both cohorts. In our cognition cohort, the median age was 56.0 (IQR, 50.0–61.0) and 51.3% were female. 2,971 (17.8%) participants were diagnosed with a previous infection at least 5 years prior to baseline. These infections included 23 (0.1%) sepsis, 45 (0.3%) pneumonia, 1,681 (10.1%) other LRTIs, 674 (4.0%) UTIs and 532 (3.2%) SSTIs. 6 participants (0.0%) had multiple infection diagnoses on the same date from different infection sites. 2,770 participants (93.2%) had GP-recorded infections and 201 (6.8%) had hospital-recorded infections.

**Table 2:** Baseline characteristics of participants included in the study with data on cognitive function measures and neuroimaging outcomes, stratified by history of common infections.

Characteristics	Cohort with cognitive function measures (*N* = 16,728)		Cohort with neuroimaging measures (*N* = 14,712)	
	No infection (*N* = 13,757)	Any infection (*N* = 2971)	No infection (*N* = 12,277)	Any infection (*N* = 2,435)
Mean age at baseline assessment (years)	55.4 (7.5)	56.4 (7.4)	54.7 (7.5)	55.5 (7.5)
Median age at baseline assessment (years)	56.0 (49.0–61.0)	57.0 (51.0–62.0)	55.0 (49.0–61.0)	56.0 (49.0–61.0)
Age category (years)				
40–44	1415 (10.3%)	235 (7.9%)	1442 (11.7%)	238 (9.8%)
45–49	2054 (14.9%)	404 (13.6%)	1963 (16.0%)	373 (15.3%)
50–54	2410 (17.5%)	477 (16.1%)	2365 (19.3%)	416 (17.1%)
55–59	3056 (22.2%)	665 (22.4%)	2716 (22.1%)	551 (22.6%)
60–64	3279 (23.8%)	769 (25.9%)	2648 (21.6%)	583 (23.9%)
65+	1543 (11.2%)	421 (14.2%)	1143 (9.3%)	274 (11.3%)
Women	6856 (49.8%)	1720 (57.9%)	6318 (51.5%)	1463 (60.1%)
Ethnicity				
White European	13,417 (97.5%)	2906 (97.8%)	11,910 (97.0%)	2365 (97.1%)
South Asian	90 (0.7%)	20 (0.7%)	110 (0.9%)	25 (1.0%)
African or Caribbean	59 (0.4%)	17 (0.6%)	61 (0.5%)	15 (0.6%)
Mixed or Other	155 (1.1%)	22 (0.7%)	158 (1.3%)	27 (1.1%)
Missing	36 (0.3%)	6 (0.2%)	38 (0.3%)	<5
Diabetes category				
No diabetes	12,229 (88.9%)	2533 (85.3%)	11,062 (90.1%)	2141 (87.9%)
Pre-diabetes	276 (2.0%)	94 (3.2%)	220 (1.8%)	59 (2.4%)
Undiagnosed diabetes	891 (6.5%)	213 (7.2%)	721 (5.9%)	163 (6.7%)
Controlled diabetes	267 (1.9%)	95 (3.2%)	205 (1.7%)	51 (2.1%)
Uncontrolled diabetes	94 (0.7%)	36 (1.2%)	69 (0.6%)	21 (0.9%)
Educational attainment (years in full-time education)	16.7 (4.3)	16.2 (4.5)	16.9 (4.3)	16.2 (4.5)
Baseline BMI (mean)	26.6 (4.2)	27.5 (4.9)	26.4 (4.1)	27.1 (4.6)
Townsend deprivation score (mean)	−2.2 (2.5)	−2.1 (2.5)	−2.1 (2.6)	−2.0 (2.6)
Baseline number of days/week moderate physical activity >10 min	3.0 (2.0–5.0)	3.0 (2.0–5.0)	3.0 (2.0–5.0)	3.0 (2.0–5.0)
Smoking status				
Never Smoker	9994 (72.6%)	2066 (69.5%)	8989 (73.2%)	1681 (69.0%)
Ex-Smoker	2931 (21.3%)	719 (24.2%)	2548 (20.8%)	590 (24.2%)
Current smoker	820 (6.0%)	181 (6.1%)	729 (5.9%)	160 (6.6%)
Missing	12 (0.1%)	5 (0.2%)	11 (0.1%)	<5
Baseline alcohol intake frequency				
Rarely or never	1752 (12.7%)	440 (14.8%)	1510 (12.3%)	350 (14.4%)
1–8 times per month	5036 (36.6%)	1123 (37.8%)	4497 (36.6%)	902 (37.0%)
16 times per month-every day	6968 (50.7%)	1406 (47.3%)	6266 (51.0%)	1181 (48.5%)
Missing	<5	<5	<5	<5
Comorbidities				
Anxiety and depression	1494 (10.9%)	474 (16.0%)	1262 (10.3%)	398 (16.3%)
Severe mental illness	149 (1.1%)	38 (1.3%)	126 (1.0%)	29 (1.2%)
Inflammatory bowel disease	519 (3.8%)	189 (6.4%)	462 (3.8%)	149 (6.1%)
Multiple Sclerosis	34 (0.2%)	17 (0.6%)	30 (0.2%)	11 (0.5%)
Rheumatoid Arthritis	112 (0.8%)	43 (1.4%)	94 (0.8%)	32 (1.3%)
Psoriasis	362 (2.6%)	98 (3.3%)	319 (2.6%)	83 (3.4%)
Asthma	1511 (11.0%)	588 (19.8%)	1380 (11.2%)	455 (18.7%)
Chronic kidney disease	112 (0.8%)	29 (1.0%)	100 (0.8%)	19 (0.8%)
Chronic liver disease	359 (2.6%)	120 (4.0%)	249 (2.0%)	79 (3.2%)
Chronic obstructive pulmonary disease	44 (0.3%)	70 (2.4%)	28 (0.2%)	47 (1.9%)
Heart failure	143 (1.0%)	65 (2.2%)	113 (0.9%)	36 (1.5%)
Hypertension	2020 (14.7%)	593 (20.0%)	1574 (12.8%)	408 (16.8%)
Obstructive sleep apnoea	86 (0.6%)	40 (1.3%)	77 (0.6%)	20 (0.8%)
Stroke	88 (0.6%)	28 (0.9%)	58 (0.5%)	22 (0.9%)
Traumatic brain injury	88 (0.6%)	30 (1.0%)	67 (0.5%)	25 (1.0%)

For data protection, table cells containing fewer than 5 participants were recorded as ‘<5’.

**Fig. 2:**
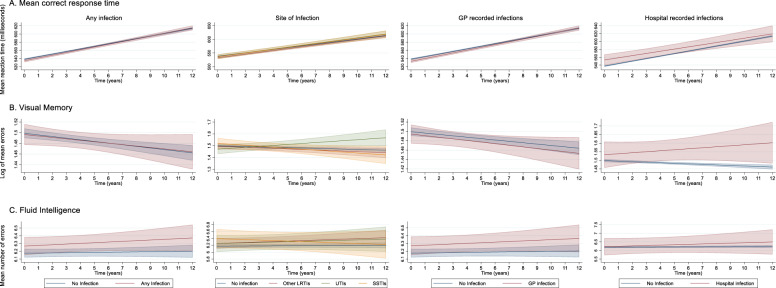
Association of presence, site and setting (GP and hospital) with changes in cognitive performance over follow-up. Linear mixed models with random intercept and slope used to illustrate fitted changes in cognitive function over time for the mean correct response time, visual memory (log transformed) and fluid intelligence test. **A** Mean correct response time models adjusted for age (years), sex, time, baseline test score, interaction term with time × infection status, ethnicity, BMI, years in full-time education, physical activity, alcohol consumption, diabetes, anxiety and depression, COPD, multiple sclerosis, hypertension and heart failure. **B** Visual memory models adjusted for age (years), sex, time, baseline test score, interaction term with time × infection status, ethnicity, BMI, smoking status, socioeconomic deprivation, physical activity, alcohol frequency, years in full-time education, diabetes, anxiety and depression, COPD, hypertension, inflammatory bowel disease, rheumatoid arthritis, obstructive sleep apnoea, and multiple sclerosis. **C** Fluid intelligence models adjusted for age (years), sex, time, baseline test score, interaction term with time × infection status and years in full-time education.

Figure 2 and Table 3 show no evidence for differences in cognitive performance change over follow-up in participants with a history of any infections compared to those without infections for the mean correct response time (estimated difference in slope [infections versus no infections] = 0.40 ms, 95% CI: −0.17–0.96 per year), visual memory (estimated difference in slope 0.0004 log errors per year, 95% CI: (−0.003–0.004), fluid intelligence (estimated difference in slope 0.007, 95% CI: −0.010–0.023) and prospective memory tests (OR 0.88, 95% CI: 0.68–1.14). The results for all covariates for these models are shown in Supplementary Table 3. No evidence of an association was found between the site and clinical setting of infections with cognitive decline for any of the tests, apart from visual memory. The log of the visual memory errors increased by 0.011 (95% CI: 0.004–0.018) per year in participants with a history of UTIs compared to those with no prior infection.

**Table 3:** Association of site and clinical setting of common infections with cognitive decline.

	**Minimally adjusted**			**Fully adjusted model**		
	**No. of participants**	**β (95% CI)**	***P*** **value**	**No. of participants**	**β (95% CI)**	***P*** **value**
Mean correct response time (Difference in slope compared with no infection)						
Site of infection						
No infection	13,707	Reference		13,275	Reference	
Any infection	2956	0.47 (−0.09 to 1.03)	0.10	2809	0.40 (−0.17 to 0.96)	0.17
LRTIs	1682	0.50 (−0.21 to 1.22)	0.17	1598	0.34 (−0.39 to 1.07)	0.37
UTIs	672	0.39 (−0.70 to 1.47)	0.49	636	0.57 (−0.54 to 1.68)	0.31
SSTI	529	0.63 (−0.58 to 1.84)	0.31	507	0.53 (−0.70 to 1.75)	0.40
Clinical setting of infection						
No infection	13,906	Reference		13,463	Reference	
GP infection	2757	0.56 (−0.02 to 1.13)	0.06	2621	0.50 (−0.09 to 1.08)	0.10
No infection	16,464	Reference		15,896	Reference	
Hospital-recorded infections	199	−0.62 (−2.50 to 1.27)	0.52	188	−0.83 (−2.76 to 1.09)	0.40
Visual memory (Difference in slope compared with no infection)						
Site of infection						
No infection	11,873	Reference		11,481	Reference	
Any infection	2562	0.00 (−0.00 to 0.00)	0.73	2436	0.00036 (−0.0034 to 0.0041)	0.85
LRTIs	1461	−0.0016 (−0.0063 to 0.00)	0.52	1387	−0.0015 (−0.0064 to 0.0033)	0.53
UTIs	579	0.011 (0.0040 to 0.018)	0.002	548	0.011 (0.0037 to 0.018)	0.003
SSTI	459	−0.0040 (−0.012 to 0.0040)	0.33	441	−0.0051 (−0.013 to 0.0030)	0.22
Clinical setting of infection						
No infection	12,041	Reference		11,639	Reference	
GP infection	2394	−0.00029 (−0.0041 to 0.0035)	0.88	2278	−0.00049 (−0.0044 to 0.0034)	0.80
No infection	14,267	Reference		13,759	Reference	
Hospital-recorded infections	168	0.010 (−0.0024 to 0.022)	0.11	158	0.0089 (−0.0039 to 0.022)	0.17
Fluid intelligence (Difference in slope compared with no infection)						
Site of infection						
No infection	4685	Reference		4673	Reference	
Any infection	1070	0.0063 (−0.010 to 0.023)	0.46	1066	0.0066 (−0.010 to 0.023)	0.44
LRTIs	619	0.012 (−0.0091 to 0.033)	0.27	616	0.011 (−0.0092 to 0.033)	0.27
UTIs	245	0.0083 (−0.025 to 0.041)	0.62	244	0.0086 (−0.024 to 0.042)	0.61
SSTI	184	−0.018 (−0.055 to 0.019)	0.34	184	−0.0162 (−0.053 to 0.021)	0.39
Clinical setting						
No infection	4743	Reference		4731	Reference	
GP infection	1012	0.0051 (−0.012 to 0.022)	0.56	1008	0.0055 (−0.012 to 0.023)	0.53
No infection	5697	Reference		5681	Reference	
Hospital-recorded infections	58	0.021 (−0.044 to 0.085)	0.53	58	0.020 (−0.044 to 0.084)	0.55
**Prospective memory**	**No. of participants**	**OR (95% CI)**	***P*** **value**	**No. of participants**	**OR (95% CI)**	***P*** **value**
Site of infection						
No infection	4174	Reference		4,083	Reference	
Any infection	926	0.83 (0.65 to 1.06)	0.14	894	0.88 (0.68 to 1.14)	0.33
LRTIs	549	072 (0.54 to 0.97)	0.03	532	0.76 (0.56 to 1.03)	0.07
UTIs	204	0.82 (0.51 to 1.33)	0.42	198	0.88 (0.53 to 1.46)	0.63
SSTI	154	1.58 (0.77 to 3.26)	0.21	147	1.74 (0.80 to 3.75)	0.16
Clinical setting						
No infection	4221	Reference		4,127	Reference	
GP infection	879	0.82 (0.64 to 1.06)	0.13	850	0.88 (0.68 to 1.14)	0.33
No infection	5053	Reference		4933	Reference	
Hospital infection	47	1.07 (0.38 to 2.99)	0.90	44	0.95 (0.34 to 2.69)	0.93

Linear Mixed models results with random intercept and random slope. The associations of site of infection, GP infection and hospital infection were not assessed in the same model but rather in three separate models. For analyses on site of infections, sepsis and pneumonia were not included due to a small number of infections (23 and 45 participants, respectively). For mean correct response time, visual memory (log transformed) and fluid intelligence tests, minimally adjusted: age (years), sex, time, baseline test score and time × infection status interaction term which represents the rate of decline by presence of infection with the difference in slope compared to that of no infection (reference group). For mean correct response time, fully adjusted models additionally adjusted for ethnicity, BMI, years in full-time education, physical activity, alcohol consumption, diabetes, anxiety and depression, COPD, multiple sclerosis, hypertension and heart failure. For the visual memory test, fully adjusted models additionally adjusted for ethnicity, BMI, smoking status, socioeconomic deprivation, physical activity, alcohol frequency, years in full-time education, diabetes, anxiety and depression, COPD, hypertension, inflammatory bowel disease, rheumatoid arthritis, obstructive sleep apnoea, and multiple sclerosis. For the fluid intelligence test, fully adjusted models additionally included years in full-time education. For the prospective memory test logistic regression was performed and the estimates reported are odds ratios. Minimally adjusted models for this test include age (years) and sex and fully adjusted models additionally adjusted for physical activity in the fully adjusted models.

### Association between infections and neuroimaging outcomes

**Fig. 3:**
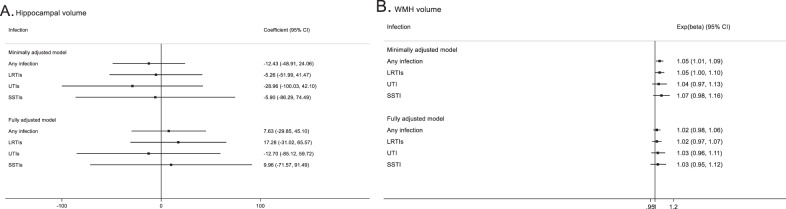
Association of presence and site of common infections with hippocampal volume and WMH volume. WMH; White matter hyperintensities. **A** represents the association between common infections and hippocampal volume. Minimally adjusted models for adjusted for age (years) and sex. Fully adjusted models additionally adjusted for ethnicity, BMI, smoking, physical activity, alcohol consumption, years in full-time education, diabetes, chronic obstructive pulmonary disease, asthma and hypertension (*n* = 14,239). **B** represents the association of common infections with WMH volume. Minimally adjusted models for (**B**) adjusted for age (years) and sex and fully adjusted models additionally adjusted for BMI, smoking and hypertension (14,357).

Figure 3 shows that a history of any infections and LRTIs excluding pneumonia was associated with a 5% higher WMH volume compared to no prior infection in minimally adjusted models. In fully adjusted analyses, the difference reduced to a 2% higher WMH volume for those with infections and included the null. No evidence of an association was found between the presence or site of infections with hippocampal volume in minimally or fully adjusted models.

### Additional secondary analyses and sensitivity analyses

Secondary analyses on the association between numbers of infections and cognitive decline show that the increase over time in the log of visual memory errors was slightly steeper by 0.006 (95% CI: 0.002 to 0.011) per year, for every additional infection (Supplementary Table 4). No evidence of an association was found between the number of infections beyond the first and cognitive decline in all other tests. Supplementary Fig. 2 depicts the association between the timing of infections in the 5 years prior to baseline and cognitive decline in fully adjusted models. We found no evidence of an association between common infections in each year prior to baseline and cognitive decline according to any of the tests. In further additional secondary analyses, we found evidence of an interaction by diabetes for mean correct response time (*p* for interaction = 0.015). Participants with diabetes and infections performed better on mean correct response time compared to participants with diabetes and no infection. In stratified results, there was no evidence of an association between common infections and cognitive decline in any of the cognitive tests in people with and without diabetes (Supplementary Table 5). We found no difference by sex and age in the association between infections and cognitive decline for any of the cognitive measures (Supplementary Tables 6, 7). Our sensitivity analyses did not materially change our conclusions (Supplementary Tables 8–12).

## Discussion

To our knowledge, this is the largest longitudinal study to date examining the association of common infections occurring in midlife with subclinical markers of dementia risk (cognitive decline, hippocampal volume and WMH volume). We did not find evidence for an association between common infections and cognitive decline for any of the cognitive measures, except for visual memory. We found evidence of small associations between UTIs and an increasing number of infections with worsening of performance on the visual memory test over follow-up. Infections were not associated with lower hippocampal or higher WMH volume after adjustments for potential confounding.

### Previous studies

Previous studies have yielded mixed findings for the association between infections and cognitive impairment. In a US case-control study of intensive care unit survivors (ICU) with a mean age of 65.9 years, no association was found between sepsis and cognitive decline [42]. However, given that ICU stay and sepsis are associated with mortality risk, the competing risk of mortality in ICU survivors with sepsis may have weakened the associations found in this study [43, 44]. Two prospective US studies with a mean and median age, respectively, of 77 years found an association between pneumonia or severe sepsis hospitalisation and cognitive impairment [10, 12].

Differences in our findings compared to previous studies may be due to heterogeneity in populations studied including their ages, clinical setting, site of infection, cognitive measures and domains assessed, and study design. Participants in our study were younger (mean/median both 56 years) than those in previous studies. Age is an important risk factor for cognitive decline and dementia, with the risk of dementia doubling every 5 years after the age of 65 [45]. We found no evidence of an association between hospitalised infections with cognitive decline, however, we had a reduced power to detect an association as only a small subset of 201 (6.8%) participants with infections had hospitalised infections while those with hospital infections in previous studies ranged from 827 to 1,529 participants [10, 12, 42]. These studies only assessed sepsis or pneumonia as individual exposures; however, our study had insufficient statistical power to study the association of these infections with cognitive decline individually. Given that exposed participants in our study were predominantly diagnosed with more mild infections (GP-recorded infections and other LRTIs), this may explain the lack of association observed as it is hypothesised that more severe systemic infections may be more likely to trigger the release of pro-inflammatory cytokines and induce systemic inflammation (or other pathways) which may lead to cognitive decline or dementia [46]. Alternatively, it is also possible that hospitalised infections do not play a role in cognitive decline and the association shown by previous studies may be driven by factors related to hospitalisation and cognition.

Two case-control studies found lower hippocampal volume in hospitalised individuals with sepsis compared to healthy controls [41, 47]. These studies were limited by their small sample size (<45 participants each) and one study had inadequately matched controls. Differences in study design and statistical method limited comparability of these studies with our findings. White matter lesions have been identified in septic shock patients who developed acute brain dysfunction, however, there is a lack of studies examining the association of common, predominantly bacterial infections, other than sepsis with WMH or hippocampal volume [48].

An explanation for our lack of evidence of an association between infections, cognitive decline, hippocampal or WMH volume could be that these neuroimaging or cognitive measures may have lacked sufficient sensitivity to detect subtle brain changes following infection in the prodromal phases. Alternatively, it could be that our finding of small associations between infections and cognitive decline on the visual memory test should be interpreted with caution. Given the number of analyses on multiple cognitive tests conducted in this study, it is possible that these findings may have occurred by chance.

### Strengths and limitations

Strengths of this study include the large size of the study population and the use of repeated assessments over follow-up assessing four individual domains of cognition. The extensive information on sociodemographic and lifestyle factors and comorbidities linked to primary and secondary care electronic health records allowed for the adjustment of multiple confounding variables. Furthermore, we explored medically recorded common infections by infection site, severity, timing and dose response as well as conducting extensive secondary and sensitivity analyses.

Our study had several limitations. First, there was evidence of a ‘healthy volunteer’ bias in the cohort studied and in the overall UK Biobank population [25]. In this study, this selection bias was likely given that participants with poorer cognitive ability and fewer years in education were more likely to have no follow-up cognitive measures. This selective attrition would likely underestimate any associations of common infections with cognitive decline thus biasing our estimates towards the null. Selection bias may have also occurred if individuals with more severe infections did not attend the baseline assessment, biasing associations towards the null. Second, our findings may also have been influenced by a potential practice effect in which participants’ performance on the same cognitive tests improved during follow-up due to familiarity with the test [49]. Although both infected and uninfected groups would have been expected to have a similar benefit of practice, these practice effects may dilute any differences between the two groups therefore potentially masking any association of infections on cognitive decline [31, 49]. Third, cognitive tests were non-standardised and visual memory test results have poor correlation between the baseline and the first repeat assessment (*r* = 0.16) which will likely have led to non-differential measurement error, biasing effect estimates towards the null [31]. Fourth, although we assessed a wide range of potential confounders, we cannot rule out the possibility of residual confounding through unmeasured confounders e.g. frailty and other forms of physical activity such as light intensity physical activity or social activities. Fifth, we restricted our analyses to only infections diagnosed within 5 years prior to baseline, therefore, infections occurring prior to this period were not included in our primary analyses which may underestimate our associations. Sixth, neuroimaging measures were assessed at one time point in our study thus we were unable to investigate longitudinal changes in hippocampal and WMH volume over time. Seventh, our study focused on a population without dementia or cognitive impairment at baseline as such our findings cannot be generalised to individuals with existing cognitive impairment/dementia. Eighth, participants in our study had a mean age of ~55 years old which may limit the generalisability of our findings to older adults, who are at a greater risk of cognitive decline and dementia. Lastly, our study population was healthier than the general population and had more years in education compared to those excluded during follow-up. This may have delayed cognitive decline thus reducing the ability to detect a significant association between common infections and cognitive decline.

## Conclusion

In summary, our findings extend the scarce literature on infections, cognitive decline and neuroimaging measures into a younger age group than the majority of previous studies. We found no evidence of accelerated cognitive decline in people across the age groups of 40–69 (at UK Biobank recruitment) with a history of common infections occurring in midlife compared to those without infections in all cognitive tests except for visual memory. Further studies are needed to assess the potential effects of common infections on cognition at different life stages and in more representative populations, including ethnic and other diversity with sufficient sample sizes to test different types of infection. These studies need careful attention to loss to follow-up as well as incorporating the impacts of mortality. Future studies should also explore trajectories of cognitive decline in people with pre-existing cognitive impairment or dementia following infection and investigate longitudinal changes in hippocampal volume, WMH volume and other subclinical neuropathologies over time in individuals with and without infections.

## Supplementary information

Supplementary Information

## Data Availability

Analysis codes used in this study can be accessed at https://github.com/RutendoMuzambi/InfectionsCognitionNeuroimaging.

## Data availability

Data are available to researchers through an application via https://www.ukbiobank.ac.uk/enable-your-research/register.
