# Supplementary material for: Are infections associated with cognitive decline and neuroimaging outcomes? A historical cohort study using data from the UK Biobank study linked to electronic health records
Source: Transl Psychiatry. 2022 Sep 15;12:385. doi: 10.1038/s41398-022-02145-z (PMC9478085; doi:10.1038/s41398-022-02145-z)
Supplement: Supplementary file 1 — Supplementary Information [file 41398_2022_2145_MOESM1_ESM.docx]

**Supplementary methods and results**

Are infections associated with cognitive decline and neuroimaging outcomes? A historical cohort study using data from the UK Biobank study linked to electronic health records

**Authors:** Rutendo Muzambi^1^, Krishnan Bhaskaran^1^, Christopher T. Rentsch^12^, Liam Smeeth^1^, Carol Brayne^3^, Victoria Garfield^4^, Dylan M. Williams^45^, Nish Chaturvedi^4^, Charlotte Warren-Gash^1^

**Contents**

**Appendix S1: supplementary methods**

**Appendix S2: supplementary Tables and Figures**

**Supplementary Table 1.** Years of schooling using UK Biobank qualifications and ISCED 1997

**Supplementary Table 2:** Infection profile of participants included and excluded from the study (cognition cohort)

**Supplementary table 3.** Association of any common infections with cognitive decline (models showing output of all included covariates)

**Supplementary Table 4.** Association of frequency of common infections with cognitive decline

**Supplementary Table 5:** Association of common infections on cognitive decline, stratified by diabetes status

**Supplementary Table 6:** Association of common infections with cognitive decline, stratified by sex

**Supplementary table 7:** Association of common infections with cognitive decline, stratified by age

**Supplementary Table 8:** Association of common infections with cognitive decline using inverse transformed reaction time

**Supplementary Table 9:** Association of common infections with cognitive decline, with at least 5 years registration in GP records

**Supplementary Table 10:** Association of common infections with cognitive decline, excluding follow up infections

**Supplementary Table 11:** Association of common infections with hippocampal volume and white matter hyperintensities volume, excluding follow up infections

**Supplementary Table 12:** Association of common infections with left and right

**Supplementary Figure 1.** Percentage of participants with and without infections stratified by age in the cognitive decline and neuroimaging cohort

**Supplementary Figure 2.** Association of common infections and cognitive decline, stratified by timing of common infections in the five years prior to baseline

**Appendix S3: study protocol**

**Supplementary Methods**

**UK Biobank study population**

9, 238,453 million individuals registered with the National Health Service who lived within 40 km of one of the 22 UK Biobank assessment centres in England, Scotland and Wales were invited to take part in the UKB study via postal invitations. Of these individuals, the response rate was low with 503, 317 (5.5%) participating in the baseline assessment which took place between 2006 and 2010.[1] During the baseline assessment visit, participants signed consent to the study, completed a web-based touch screen questionnaire that assessed medical history, lifestyle and behavioural factors, and sociodemographic factors. Participants attended a nurse interview, completed cognitive tests, underwent physical examinations and provided biological samples (blood, urine and saliva collections).[2]

UK biobank data was linked to routinely available national databases including hospital admission, death registry and primary care records. Approximately 230,000 participants (45%) of the total UK Biobank population provided written consent for linkage of the primary care records. [3] Data was collected from GP practices in England, Scotland and Wales using EMIS, Vision and TPP GP computer system suppliers. Diagnoses were recorded using Read v2 or Clinical Terms Version 3 (CTV3) coding classification. Prescription data from these practices was coded using Read v2, British National Formulary or Dictionary of Medicines and Devices (dm+d) coding classifications.

Our study included participants with linked primary and secondary care data. We excluded participants who had less than 12 months registration with a GP practice to avoid incorporating historical diagnoses when defining our exposure.

**Cognitive function measures**

Participants undertook a brief 15-minute cognitive test battery to assess cognitive function using a touchscreen computer. Participants were given instructions for the test on the screen and completed the assessment without supervision. These tests included reaction time (mean correct response time), visual memory, fluid intelligence and prospective memory. The tests are described in detail by Lyall et al, 2016.[4]

When calculating the mean correct response time, rounds 0-4 were regarded as “training” so were excluded in the present study in accordance to the approach employed by the UK Biobank. To deal with outliers, we excluded reaction times under 50 milliseconds and over 2000 milliseconds.[5] We acknowledge that there are other approaches to dealing with reaction time outliers using cut-offs based a number of standard deviations above the mean response time.[6]

**Neuroimaging measures**

Structural MRI scans included in the UK Biobank protocol were T1, T2 fluid attenuation inversion recovery (FLAIR), susceptibility-weighted MRI, diffusion MRI and resting and task functional MRI. To ascertain the volume of white matter hyperintensities, we used T1 and T2 FLAIR images. T1 scans, which allow precise volumetric measures of cortical, subcortical and whole brain regions, were also used to determine the volume of the left and right hippocampus separately. These subcortical structures were modelled using FMRIB’s Integrated Registration and Segmentation Tool (FIRST).[7, 8]

**Follow up cognitive function and neuroimaging data**

Between August 2012 and June 2013, a subset of approximately 20,000 participants who lived within a 35km radius of the Stockport coordinating centre were invited via email to attend a repeat assessment of the UK Biobank baseline measures. Repeat assessments were conducted on cognitive function measures as well as other information including that pertaining to health and lifestyle information, and physical measurements. [9] The second repeat follow-up targeting the same regions which also assessed cognitive function began in 2014. In 2014, participants completed the fluid intelligence or pairs matching tests online or at the assessment centre. In our study, we only included participants who conducted the tests at the assessment centre.

From April 2014, participants were re-invited via email to undergo magnetic resonance imaging including brain imaging. Imaging examinations took place at assessment centres in Stockport, Newcastle-upon-Tyne, Reading and Bristol. These centres were chosen in order to limit travel times for the majority of participants. [8] Quality assurance across all imaging centres was managed through a centralised training and monitoring team. A six-week training programme was attended by all staff members prior to the opening of centres and monthly training was provided by the MR physicist. Across all centres, a standardised training programme for all radiographers, standard operating procedures and other quality assurance and control measures were employed. Identical protocols, scanner models, software, types of coils and adjustment and tuning methods were used in each centre to ensure fully harmonised imaging data.[8]

All participants included in our study had completed baseline cognitive function assessment and at least one follow up assessment for the same test. For each test, some participants attended only one follow up assessment and some attended both follow up assessments.[4, 10] The fluid intelligence and prospective memory were added part way through the baseline assessment and only used at ten assessment centres as such baseline data on these tests were missing for the majority of participants. Other tests such as the numeric memory test were not included in the present study as they were removed during the baseline assessment and not included in the first repeat assessment. [4] Fluid intelligence and reaction time had an adequate test retest reliability of 0.65 and 0.54, respectively, while the visual memory test had a poor test retest reliability of 0.16 between the baseline and the first repeat assessment. [4] The poor reliability is likely to bias any effect estimates to the null.

**Infections**

For our ever infection exposure, if participants were diagnosed with more than one infection during this period, the infection date was taken from the earliest record of infection.

For analyses on frequency of infection, infections diagnosed within 28 days of each other were classified as a single episode of infection. Secondary analyses examined the frequency of infections on cognitive decline.

**Covariates**

Demographic variables included age (years), sex, ethnicity (white, south Asian, black, mixed or other) and education. Education was defined as years in full-time education using qualifications based on the International Standard Classification of Education (ISCED) 1997 (Supplementary Table 1).[11, 12] Socioeconomic status was measured using the Townsend Deprivation scores, based on residential post codes at baseline.[13] Potential lifestyle factors included body mass index (BMI, kg/m^2^), smoking (never, former, or current ), alcohol intake frequency (rarely or never, 1-8 times per month or 16 times per month) and physical activity (number of days a week where participants spent >10 minutes of moderate physical activity).

**Socioeconomic deprivation**

The Townsend deprivation index at recruitment (data field 189), which was used as a proxy for socioeconomic status.[14] The Townsend deprivation score was assigned to each participant using postcodes and is calculated from unemployment, non-car ownership, non-home ownership and household overcrowding data.[13] Positive scores (greater than zero) represent higher than average deprivation and negative scores (below zero) represent less deprivation/relative affluence.

**Education attainment (years in full time education)**

Baseline qualifications were used to ascertain the years in full-time education of the participants. We used the International Standard Classification of Education ISCED 1997 (ISCED 1997) and applied the classification to the UK’s educational qualifications.[11, 12] Participants who responded with “prefer not to answer” were coded as missing.

**Comorbidities**

All comorbidities were ascertained using data from baseline assessment questionnaires, nurse interview and linked primary and secondary care data. In electronic health records covariates were defined within 10 years prior to baseline.

**Missing data**

3.6% (n=595) of the cognition cohort study population had missing data on ethnicity (n=42), BMI (n=40), years in full-time education (n=65), alcohol consumption (n=<5), smoking (n=17) physical activity (n=447), Townsend deprivation index (n=10). In total, 3.2% (n=469) of the neuroimaging cohort had missing data on ethnicity (n=41), BMI (n=28), years in full-time education (n=53), alcohol consumption (n=6), smoking (n=15), physical activity (n=359) and Townsend deprivation index (n=8). Due to the small proportion of missing data in both cohorts and the fact that not all covariates were used in all analyses, we used a complete case analysis.

**Supplementary Table S1. Years of schooling using UK Biobank qualifications and ISCED 1997**

| **UK Biobank**  **coding** | **UK Biobank qualifications (data field 6138)** | **ISCED 1997**  **level** | **Years of**  **schooling** |
| --- | --- | --- | --- |
| -7 | None of the above | 1 | 7 |
| -3 | Prefer not to answer | - | - |
| 1 | College or University degree | 5 | 20 |
| 2 | A levels/AS levels or equivalent | 3 | 13 |
| 3 | O levels/GCSEs or equivalent | 2 | 10 |
| 4 | CSEs or equivalent | 2 | 10 |
| 5 | NVQ or HND or HNC or equivalent | 5 | 19 |
| 6 | Other professional qualifications eg: nursing, teaching | 4 | 15 |

**Statistical analyses**

Linear Mixed models

We estimated the association between common infections and cognitive changes over follow up using linear mixed models with random intercept and slope effects, estimated by restricted maximum likelihood and using an unstructured covariance matrix. Linear mixed models were chosen as they account for the correlation of repeated measures over time, use all available data over follow up and can handle missing data.

Using Q-Q plots, we found that the distribution of residuals for mean correct response time was right skewed. When we log transformed response time, the distribution of residuals still appeared right skewed, though more normally distributed than the raw mean correct response time variable. We then inverse transformed the raw mean correct response time variable and the residuals appeared normally distributed. However, models with the inverse or log transformed variable either failed to provide standard errors or failed to converge when adding covariates into the model. As a result, we ran our main analysis using raw mean correct response time and then a sensitivity analysis. In this sensitivity analysis, we repeated our main analysis using the inverse transformed response time but specified a model using a simple covariance structure matrix (independent instead of unstructured). The drawback of this model is that it assumes that observations on the same person over time are independent and are thus not correlated.

**Supplementary table 2: Infection profile of participants included and excluded from the study (cognition cohort)**

| **Characteristic** | **Included cohort (N=2,971)** | **Cohort with no follow up cognitive measures (N=31,381)** |
| --- | --- | --- |
| **Infection site** |  |  |
| Other Lower respiratory tract infections | 1,691 (56.9%) | 19,073 (60.8%) |
| UTI | 674 (22.7%) | 6,089 (19.4%) |
| SSTI | 532 (17.9%) | 5,260 (16.8%) |
| Pneumonia | 45 ( 1.5%) | 658 ( 2.1%) |
| Sepsis | 23 ( 0.8%) | 255 ( 0.8%) |
| Multiple infections diagnosed at different sites on the same date | 6 ( 0.2%) | 46 ( 0.1%) |
| **Infection clinical setting** |  |  |
| GP infections | 2,770 (93.2%) | 28,246 (90.0%) |
| Hospital infections | 201 ( 6.8%) | 3,135 (10.0%) |
| **Frequency of infections** |  |  |
| Number of infections, mean (sd) | 1.48 (1.13) | 1.60 (1.31) |
| Infection numbers (category) |  |  |
| 1 infection | 2,154 (72.5%) | 21,522 (68.6%) |
| 2 infections | 536 (18.0%) | 5,799 (18.5%) |
| 3+ infections | 281 ( 9.5%) | 4,060 (12.9%) |
| **Mortality due to any common infection** | 5 (0.2%) | 323 (1.0%) |

**Supplementary table 3. Association of any common infections with cognitive decline (models showing output of all included covariates)**

|  | **Fully adjusted model** | | |
| --- | --- | --- | --- |
|  | **No. of participants** | **β (95% CI)** | **P value** |
| **Mean correct response time** (Difference in slope compared with no infection) | | | |
| **No infection** | 13,275 | Reference |  |
| **Any infection** | 2.809 | -3.71 (-7.64 to 0.21) | 0.06 |
| **Time** | 16,084 | 6.29 (6.06 to 6.52) | 0.000cf |
| **Any infection (interaction term with time)** | 2.809 | 0.40 (-0.17 to 0.96) | 0.17 |
| **Age (years)** | 16,084 | 3.81 (3.63 to 4.00) | 0.000 |
| **Gender** |  |  |  |
| Women | 8,197 | Reference |  |
| Men | 7,887 | -20.81 (-23.55 to -18.06) | 0.000 |
| **Ethnicity** |  |  |  |
| White European | 15,745 | Reference |  |
| South Asian | 106 | 54.94 (38.39 to 71.48) | 0.000 |
| African Caribbean | 70 | 68.88 (48.59 to 89.18) | 0.000 |
| Mixed or other | 163 | 6.65 (-6.63 to 19.93) | 0.33 |
| **BMI** | 16,084 | -0.19 (-0.51 to 0.13) | 0.25 |
| **Number of days/week moderate physical activity >10 mins** | 16,084 | 0.04 (-0.55 to 0.62) | 0.91 |
| **Alcohol intake frequency** |  |  |  |
| Rarely or never | 2,073 | Reference |  |
| 1-8 times per month | 5,926 | -0.38 (-4.70 to 3.95) | 0.86 |
| 16 times per month (every day) | 8,085 | -2.88 (-7.12 to 1.35) | 0.18 |
| **Education (years)** | 16,084 | -1.28 (-1.59 to -0.97) | 0.000 |
| **Diabetes status** |  |  |  |
| No diabetes | 14,217 | Reference |  |
| Pre-diabetes | 352 | 1.57 (-7.58 to 10.72) | 0.74 |
| Undiagnosed diabetes | 1,060 | 2.67 (-2.67 to 8.00) | 0.33 |
| Controlled diabetes | 332 | 12.90 (2.34 to 23.45) | 0.02 |
| Uncontrolled diabetes | 123 | 13.13 (-2.98 to 29.23) | 0.11 |
| **Anxiety and depression** | 1,884 | 4.28 (0.13 to 8.43) | 0.04 |
| **COPD** | 105 | 20.17 (3.66 to 36.67) | 0.02 |
| **Multiple sclerosis** | 46 | 36.03 (11.32 to 60.74) | 0.004 |
| **Hypertension** | 2,486 | 5.35 (1.46 to 9.23) | 0.001 |
| **Heart failure** | 187 | 0.32 (-14.20 to 14.84) | 0.97 |
| **Visual memory** (Difference in slope compared with no infection) | | | |
| **No infection** | 11,481 |  |  |
| **Any infection** | 2,436 | -0.0024 (-0.024 to 0.019) | 0.83 |
| **Time** | 13,917 | -0.0031 (-0.005 to -0.02) | 0.000 |
| **Any infection (interaction term with time)** | 2,436 | 0.0004 (-0.003 to 0.004) | 0.85 |
| **Age (years)** | 13,917 | 0.0084 (0.008 to 0.009) | 0.000 |
| **Gender** |  |  |  |
| Women | 7,124 | Ref |  |
| Men | 6,793 | 0.0179 (0.005 to 0.031) | 0.007 |
| **Ethnicity** |  |  |  |
| White European | 13,624 | Ref |  |
| South Asian | 91 | 0.17 (0.09 to 0.25) | 0.000 |
| African Caribbean | 60 | 0.20 (0.10 to 0.30) | 0.000 |
| Mixed or other | 142 | 0.12 (0.05 to 0.18) | 0.000 |
| **BMI** | 13,917 | -0.0042 (-0.06 to -0.03) | 0.000 |
| **Smoking status** |  |  |  |
| Never smoker | 10,060 | Ref |  |
| Previous smoker | 3,041 | -0.0275 (-0.043 to -0.012) | 0.001 |
| Current smoker | 816 | 0.0075 (-0.020 to 0.035) | 0.59 |
| **Townsend deprivation score (mean)** | 13,917 | 0.0033 (0.001 to 0.006) | 0.01 |
| **Baseline number of days/week moderate physical activity >10 mins** | 13,917 | 0.0045 (0.002 to 0.007) | 0.001 |
| **Alcohol intake frequency** |  |  |  |
| Rarely or never | 1,819 | Reference |  |
| 1-8 times per month | 5,119 | -0.0181 (-0.038 to 0.002) | 0.08 |
| 16 times per month (every day) | 6,979 | -0.0221 (-0.042 to -0.002) | 0.03 |
| **Education (years)** | 13,917 | -0.0025 (-0.004 to -0.001) | 0.001 |
| **Diabetes status** |  |  |  |
| No diabetes | 12,284 | Reference |  |
| Pre-diabetes | 309 | 0.019 (-0.024 to 0.062) | 0.38 |
| Undiagnosed diabetes | 934 | -0.028 (-0.053 to -0.003) | 0.03 |
| Controlled diabetes | 284 | -0.001 (-0.046 to 0.044) | 0.96 |
| Uncontrolled diabetes | 106 | -0.030 (-0.101 to 0.042) | 0.42 |
| **Anxiety and depression** | 1,637 | 0.048 (0.028 to 0.067) | 0.000 |
| **COPD** | 96 | 0.028 (-0.104 to 0.049) | 0.48 |
| **Inflammatory bowel disease** | 595 | 0.027 (-0.004 to 0.059) | 0.09 |
| **Rheumatoid arthritis** | 126 | -0.031 (-0.098 to 0.035) | 0.35 |
| **Obstructive sleep apnoea** | 104 | 0.127 (0.053 to 0.201) | 0.001 |
| **Multiple sclerosis** | 43 | 0.065 (-0.048 to 0.177) | 0.26 |
| **Hypertension** | 2,178 | 0.018 (0.000 to 0.036) | 0.06 |
| **Fluid intelligence (Difference in slope compared with no infection)** | | | |
| **No infection** | 4,673 | Reference |  |
| **Any infection** | 1,066 | 0.092 (-0.036 to 0.219) | 0.16 |
| **Time** | 5,739 | 0.002 (-0.005 to 0.009) | 0.58 |
| **Any infection (interaction term with time)** | 1,066 | 0.007 (-0.010 to 0.023) | 0.44 |
| **Age (years)** | 5,739 | -0.004 (-0.010 to 0.002) | 0.16 |
| **Gender** |  |  |  |
| Women | 2,882 | Reference |  |
| Men | 2,857 | -0.156 (-0.247 to -0.065) | 0.001 |
| **Education (years)** | 5,739 | -0.131 (-0.142 to -0.121) | 0.000 |
| **Prospective memory** | | | |
|  | **No. of participants** | **OR (95% CI)** | **P value** |
| **No infection** | 4,083 | Reference |  |
| **Any infection** | 894 | 0.88 (0.68 to 1.14) | 0.33 |
| **Age (years)** | 4,977 | 0.96 (0.95 to 0.97) | 0.000 |
| **Gender** |  |  |  |
| Women | 2,475 | Reference |  |
| Men | 2,502 | 1.00 (0.82 to 1.23) | 0.995 |
| **Baseline number of days/week moderate physical activity >10 mins** | 4,977 | 0.96 (0.92 to 1.00) | 0.07 |

**Supplementary Table 4. Association of frequency of common infections with cognitive decline**

|  | **Minimally adjusted** | | | **Fully adjusted model** | | |
| --- | --- | --- | --- | --- | --- | --- |
|  | **No. of participants** | **β (95% CI)** | **P value** | **No. of participants** | **β (95% CI)** | **P value** |
| **Mean correct response time** (Difference in slope compared with no infection) | | | | | | |
| No infection | 14,494 | Reference |  | 14,006 | Reference |  |
| First infection | 2,169 | 0.26 (-0.37 to 0.89) | 0.42 | 2,078 | 0.13 (-0.51 to 0.77) | 0.68 |
| Second or more infections (continuous) | 817 | -0.22 (-0.91 to 0.46) | 0.52 | 760 | -0.25 (-0.96 to 0.47) | 0.50 |
| **Visual memory** (Difference in slope compared with no infection) | | | | | | |
| No infection | 12,575 | Reference |  | 12,136 | Reference |  |
| First infection | 1,860 | 0.00 (-0.00 to 0.0052) | 0.63 | 1,781 | 0.00089 (-0.0034 to 0.0051) | 0.68 |
| Second or more infections +(continuous) | 728 | 0.0059 (0.0018 to 0.010) | 0.005 | 679 | 0.0064 (0.0019 to 0.011) | 0.005 |
| **Fluid intelligence (**Difference in slope compared with no infection) | | | | | | |
| No infection | 4,975 | Reference |  | 4,961 | Reference |  |
| First infection | 780 | 0.0048 (-0.014 to 0.024) | 0.61 | 778 | 0.0052 (-0.014 to 0.024) | 0.59 |
| Second or more infections +(continuous) | 297 | -0.014 (-0.039 to 0.012) | 0.31 | 295 | -0.014 (-0.040 to 0.011) | 0.27 |
| **Prospective memory** | | | | | | |
|  | **No. of participants** | **OR (95% CI)** | **P value** | **No. of participants** | **OR (95% CI)** | **P value** |
| No infection | 4,424 | Reference |  | 4,322 | Reference |  |
| First infection | 676 | 0.78 (0.59 to 1.02) | 0.07 | 655 | 0.79 (0.60 to 1.05) | 0.10 |
| Second or more infections +(continuous) | 256 | 0.88 (0.67 to 1.16) | 0.36 | 245 | 0.89 (0.66 to 1.21) | 0.47 |
| Linear Mixed models results with random intercept and random slope. For mean correct response time, visual memory (log transformed) and fluid intelligence tests, minimally adjusted: age (years), sex, time, baseline test score and time x infection status interaction term which represents the rate of decline by presence of infection with the difference in slope compared to that of no infection (reference group). For mean correct response time, fully adjusted models additionally adjusted for ethnicity, BMI, years in full-time education, physical activity, alcohol consumption, diabetes, anxiety and depression, COPD, multiple sclerosis, hypertension and heart failure. For the visual memory test, fully adjusted models additionally adjusted for ethnicity, BMI, smoking status, socioeconomic deprivation, physical activity, alcohol frequency, years in full-time education, diabetes, anxiety and depression, COPD, hypertension, inflammatory bowel disease, rheumatoid arthritis, obstructive sleep apnoea, and multiple sclerosis. For the fluid intelligence test, fully adjusted models additionally included years in full-time education. For the prospective memory test logistic regression was performed and the estimates reported are odds ratios. Minimally adjusted models for this test include age(years) and sex and fully adjusted models additionally adjusted for physical activity in the fully adjusted models. | | | | | | |

**Supplementary table 5: Association of common infections on cognitive decline, stratified by diabetes status**

|  | **No. of Participants** | **Fully adjusted model β (95% CI)** | **P value (Likelihood test for interaction)** |
| --- | --- | --- | --- |
| **Mean correct response time** (Difference in slope compared with no infection) | | | |
| **Diabetes** | | | 0.015 |
| No infection | 338 | Reference |  |
| Any infection | 177 | -2.79 (-6.41 to 0.83) |  |
| **No diabetes** | | |  |
| No infection | 12,937 | Reference |  |
| Any infection | 2,692 | 0.48 (-0.096 to 1.05) |  |
| **Visual memory** (Difference in slope compared with no infection) | | | |
| **Diabetes** |  |  | 1.00 |
| No infection | 288 | Reference |  |
| Any infection | 102 | -0.018 (-0.039 to 0.0024) |  |
| **No diabetes** |  |  |  |
| No infection | 11,193 | Reference |  |
| Any infection | 2,334 | 0.00090 (-0.0029 to 0.0047) |  |
| **Fluid intelligence (**Difference in slope compared with no infection) | | | |
| **Diabetes** |  |  | 0.91 |
| No infection | 142 | Reference |  |
| Any infection | 49 | -0.0033 (-0.097 to 0.090) |  |
| **No diabetes** |  |  |  |
| No infection | 4,531 | Reference |  |
| Any infection | 1,017 | 0.0072 (-0.0098 to 0.024) |  |
| **Prospective memory** | | | |
| **Diabetes** |  |  | 0.68 |
| No infection | 114 | Reference |  |
| Any infection | 37 | 0.78 (0.25 to 2.44) |  |
| **No diabetes** |  |  |  |
| No infection | 3,969 | Reference |  |
| Any infection | 857 | 0.89 (0.69 to 1.16) |  |
| Linear Mixed models results with random intercept and random slope. For mean correct response time, visual memory (log transformed) and fluid intelligence tests. An interaction term was added between infection, time and diabetes. For mean correct response time, fully adjusted models adjusted for age(years), sex, time, baseline test score, interaction term with time x infection status, ethnicity, BMI, years in full-time education, physical activity, alcohol consumption, anxiety and depression, COPD, multiple sclerosis, hypertension and heart failure. For the visual memory test, fully adjusted models adjusted for age(years), sex, time, baseline test score, interaction term with time x infection status, ethnicity, BMI, smoking status, socioeconomic deprivation, physical activity, alcohol frequency, years in full-time education, anxiety and depression, COPD, hypertension, inflammatory bowel disease, rheumatoid arthritis, obstructive sleep apnoea, and multiple sclerosis. For the fluid intelligence test, fully adjusted models included age(years), sex, time, baseline test score, interaction term with time x infection status, years in full-time education. For the prospective memory test logistic regression was performed and the estimates reported are odds ratios. An interaction term was added between infection, time and diabetes. Fully adjusted models for this test included age(years), sex and physical activity in the fully adjusted models. Likelihood ratio tests comparing models with and without interaction terms with diabetes. | | | |

**Supplementary table 6: Association of common infections with cognitive decline, stratified by sex**

|  | **Minimally adjusted** | | | **Fully adjusted model** | | |
| --- | --- | --- | --- | --- | --- | --- |
|  | No. of participants | Β (95% CI) | P value | No. of participants | Β (95% CI) | P value |
| **Mean correct response time** (Difference in slope compared with no infection) | | | | | | |
| **Male** | | | | | | |
| No infection | 6,879 | Reference | | 6,687 | Reference | |
| Any infection | 1,246 | 0.08 (-0.74 to 0.91) | 0.84 | 1,200 | 0.013 (-0.82 to 0.85) | 0.98 |
| **Female** | | | | | | |
| No infection | 6,828 | Reference | | 6,588 | Reference |  |
| Any infection | 1,710 | 0.74 (-0.02 to 1.50) | 0.06 | 1,609 | 0.68 (-0.10 to 1.45) | 0.09 |
| **Visual memory** (Difference in slope compared with no infection) | | | | | | |
| **Male** | | | | | | |
| No infection | 5,929 | Reference | | 5,755 | Reference |  |
| Any infection | 1,079 | -0.0034 (-0.0091 to 0.0023) | 0.24 | 1,038 | -0.0045 (-0.010 to 0.0013) | 0.13 |
| **Female** | | | | | | |
| No infection | 5,944 | Reference | | 5,726 | Reference | |
| Any infection | 1,483 | 0.0041 (-0.00076 to 0.0089) | 0.10 | 1,398 | 0.0044 (-0.00053 to 0.0093) | 0.08 |
| **Fluid Intelligence (Difference in slope compared with no infection)** | | | | | | |
| **Male** | | | | | | |
| No infection | 2,395 | Reference | | 2,387 | Reference | |
| Any infection | 472 | 0.012 (-0.013 to 0.038) | 0.34 | 470 | 0.014 (-0.012 to 0.039) | 0.30 |
| **Female** | | | | | | |
| No infection | 2,290 | Reference |  | 2,286 | Reference |  |
| Any infection | 598 | 0.00078 (-0.021 to 0.023) | 0.95 | 596 | 0.00056 (-0.022 to 0.023) | 0.96 |
|  | **No. of participants** | **OR (95% CI)** | **P value** | **No. of participants** | **OR (95% CI)** | **P value** |
| **Prospective memory** | | | | | | |
| **Male** | | | | | | |
| No infection | 2,141 | Reference | | 2,103 | Reference | |
| Any infection | 408 | 0.88 (0.61 to 1.26) | 0.49 | 399 | 0.91 (0.63 to 1.32) | 0.62 |
| **Female** | | | | | | |
| No infection | 2,033 | Reference | | 1,980 | Reference | |
| Any infection | 518 | 0.79 (0.57 to 1.11) | 0.18 | 495 | 0.86 (0.61 to 1.22) | 0.39 |
| Linear Mixed models results with random intercept and random slope. For mean correct response time, visual memory (log transformed) and fluid intelligence tests. minimally adjusted: age(years), sex, time, baseline test score, interaction term with time x infection status allows the calculation of the rate of decline by presence of infection with no infection as the reference group. For mean correct response time, fully adjusted models additionally adjusted for ethnicity, BMI, years in full-time education, physical activity, alcohol consumption, diabetes category, anxiety and depression, COPD, multiple sclerosis, hypertension and heart failure. For the visual memory test, fully adjusted models additionally adjusted for ethnicity, BMI, smoking status, socioeconomic deprivation, physical activity, alcohol frequency, years in full-time education, diabetes status, anxiety and depression, COPD, hypertension, inflammatory bowel disease, rheumatoid arthritis, obstructive sleep apnoea, and multiple sclerosis. For the fluid intelligence test, fully adjusted models additionally included years in full-time education. For the prospective memory test logistic regression was performed and the estimates reported are odds ratios. Minimally adjusted models for this test include age(years) and sex and fully adjusted models additionally adjusted for physical activity in the fully adjusted models. | | | | | | |

**Supplementary table 7: Association of common infections with cognitive decline, stratified by age (years)**

|  | **Minimally adjusted** | | | **Fully adjusted model** | | |
| --- | --- | --- | --- | --- | --- | --- |
|  | No. of participants | Β (95% CI) | P value | No. of participants | Β (95% CI) | P value |
| **Mean correct response time** (Difference in slope compared with no infection) | | | | | | |
| **Age (40-49 years)** |  |  |  |  |  |  |
| No infection | 3,455 | Reference | | 3,372 | Reference | |
| Any infection | 638 | -0.25 (-1.21 to 0.71) | 0.60 | 614 | -0.20 (-1.18 to 0.78) | 0.69 |
| **Age (50-59 years)** |  |  |  |  |  |  |
| No infection | 5,448 | Reference | | 5,285 | Reference | |
| Any infection | 1,139 | 0.55 (-0.34 to 1.45) | 0.23 | 1,091 | 0.40 (-0.52 to 1.31) | 0.40 |
| **Age (60+ years)** |  |  |  |  |  |  |
| No infection | 4,804 | Reference | | 4,618 | Reference | |
| Any infection | 1,179 | 0.65 (-0.37 to 1.68) | 0.21 | 1,104 | 0.59 (-0.45 to 1.63) | 0.27 |
| **Visual memory** (Difference in slope compared with no infection) | | | | | | |
| **Age (40-49 years)** |  |  |  |  |  |  |
| No infection | 2,867 | Reference | | 2,786 | Reference | |
| Any infection | 518 | 0.00 (-0.00 to 0.01) | 0.52 | 496 | 0.00 (-0.01 to 0.01) | 0.65 |
| **Age (50-59 years)** |  |  |  |  |  |  |
| No infection | 4,738 | Reference | | 4,591 | Reference | |
| Any infection | 988 | 0.00 (-0.01 to 0.01) | 0.94 | 948 | -0.00 (-0.01 to 0.01) | 0.87 |
| **Age (60+ years)** |  |  |  |  |  |  |
| No infection | 4,268 | Reference | | 4,104 | Reference | |
| Any infection | 1,056 | -0.00 (-0.01 to 0.01) | 0.96 | 992 | 0.00 (-0.01 to 0.01) | 0.89 |
| **Fluid Intelligence** (Difference in slope compared with no infection) | | | | | | |
| **Age (40-49 years)** |  |  |  |  |  |  |
| No infection | 1,145 | Reference | | 1,141 | Reference | |
| Any infection | 237 | -0.01 (-0.04 to 0.03) | 0.75 | 235 | -0.01 (-0.04 to 0.03) | 0.70 |
| **Age (50-59 years)** |  |  |  |  |  |  |
| No infection | 1,844 | Reference |  | 1,841 | Reference | |
| Any infection | 415 | 0.00 (-0.02 to 0.03) | 0.85 | 414 | 0.00 (-0.02 to 0.03) | 0.74 |
| **Age (60+ years)** |  |  |  |  |  |  |
| No infection | 1,696 | Reference | | 1,691 | Reference | |
| Any infection | 418 | 0.02 (-0.01 to 0.04) | 0.29 | 417 | 0.01 (-0.01 to 0.04) | 0.30 |
|  |  | **OR (95% CI)** | **P value** |  | **OR (95% CI)** | **P value** |
| **Prospective memory** |  |  |  |  |  |  |
| **Age (40-49 years)** |  |  |  |  |  |  |
| No infection | 1,036 | Reference | | 1,020 | Reference | |
| Any infection | 219 | 0.96 (0.52 to 1.78) | 0.90 | 216 | 0.97 (0.52 to 1.79) | 0.91 |
| **Age (50-59 years)** |  |  |  |  |  |  |
| No infection | 1,677 | Reference | | 1,636 | Reference | |
| Any infection | 372 | 1.15 (0.73 to 1.81) | 0.55 | 362 | 1.12 (0.71 to 1.78) | 0.63 |
| **Age (60+ years)** |  |  |  |  |  |  |
| No infection | 1,461 | Reference | | 1,427 | Reference | |
| Any infection | 335 | 1.38 (0.97 to 1.97) | 0.08 | 316 | 1.26 (0.86 to 1.84) | 0.23 |
| Linear Mixed models results with random intercept and random slope. For reaction time, visual memory (log transformed) and fluid intelligence tests. minimally adjusted: age(years), sex, time, baseline test score, interaction term with time x infection status allows the calculation of the rate of decline by presence of infection with no infection as the reference group. For reaction time, fully adjusted models additionally adjusted for ethnicity, BMI, years in full-time education, physical activity, alcohol consumption, diabetes category, anxiety and depression, COPD, multiple sclerosis, hypertension and heart failure. For the visual memory test, fully adjusted models additionally adjusted for ethnicity, BMI, smoking status, socioeconomic deprivation, physical activity, alcohol frequency, years in full-time education, diabetes status, anxiety and depression, COPD, hypertension, inflammatory bowel disease, rheumatoid arthritis, obstructive sleep apnoea, and multiple sclerosis. For the fluid intelligence test, fully adjusted models additionally included years in full-time education. For the prospective memory test logistic regression was performed and the estimates reported are odds ratios. Minimally adjusted models for this test include age(years) and sex and fully adjusted models additionally adjusted for physical activity in the fully adjusted models. | | | | | | |

**Supplementary table 8: Association of common infections with cognitive decline using inverse transformed reaction time with an independent covariance structure**

|  |  | **Minimally adjusted** | |  | **Fully adjusted model** | |
| --- | --- | --- | --- | --- | --- | --- |
|  | **No. of participants** | **β (95% CI)** | **P value** | **No. of participants** | **β (95% CI)** | **P value** |
| **Inverse transformed mean correct response time** (Difference in slope compared with no infection) | | | | | | |
| **Site of infection** | | | | | | |
| No infection | 13,707 | Reference | | 13,275 | Reference | |
| Any infection | 2,956 | -9.34e-07 (-2.48e-06 to 6.13e-07) | 0.24 | 2,809 | -7.63e-07 (-2.34e-06 to 8.15e-07) | 0.34 |
| Linear Mixed models results with random intercept and random slope and an independent covariance structure. Minimally adjusted model included: age(years), sex, time, baseline test score, interaction term with time x infection status allows the calculation of the rate of decline by presence of infection with no infection as the reference group. Fully adjusted models additionally adjusted for ethnicity, BMI, years in full-time education, physical activity, alcohol consumption, diabetes category, anxiety and depression, COPD, multiple sclerosis, hypertension and heart failure. | | | | | | |

**Supplementary table 9: Association of common infections with cognitive decline, with at least 5 years registration in GP records**

|  |  | **Minimally adjusted** | |  | **Fully adjusted model** | |
| --- | --- | --- | --- | --- | --- | --- |
|  | **No. of participants** | **β (95% CI)** | **P value** | **No. of participants** | **β (95% CI)** | **P value** |
| **Mean correct response time** (Difference in slope compared with no infection) | | | | | | |
| No infection | 11,562 | Reference | | 11,202 | Reference | |
| Any infection | 2,740 | 0.40 (-0.18 to 0.99) | 0.18 | 2,604 | 0.32 (-0.28 to 0.91) | 0.29 |
| **Visual memory** (Difference in slope compared with no infection) | | | | | | |
| No infection | 10,023 | Reference | | 9,703 | Reference | |
| Any infection | 2,378 | 0.00044 (-0.0034 to 0.0043) | 0.82 | 2,261 | -0.00016 (-0.0041 to 0.0038) | 0.94 |
| **Fluid intelligence** (Difference in slope compared with no infection) | | | | | | |
| No infection | 4,033 | Reference | | 4,022 | Reference | |
| Any infection | 1,005 | 0.0046 (-0.013 to 0.022) | 0.61 | 1,002 | 0.0052 (-0.012 to 0.023) | 0.56 |
|  | **No. of participants** | **OR (95% CI)** | **P value** | **No. of participants** | **OR (95% CI)** | **P value** |
| **Prospective memory** | | | | | | |
| No infection | 3,592 | Reference | | 3,515 | Reference | |
| Any infection | 872 | 0.83 (0.64 to 1.07) | 0.15 | 843 | 0.86 (0.66 to 1.12) | 0.27 |
| Linear Mixed models results with random intercept and random slope For reaction time, visual memory (log transformed) and fluid intelligence tests, minimally adjusted: age(years), sex, time, baseline test score and time x infection status interaction term which represents the rate of decline by presence of infection with the difference in slope compared to that of no infection (reference group). For reaction time, fully adjusted models additionally adjusted for ethnicity, BMI, years in full-time education, physical activity, alcohol consumption, diabetes category, anxiety and depression, COPD, multiple sclerosis, hypertension and heart failure. For the visual memory test, fully adjusted models additionally adjusted for ethnicity, BMI, smoking status, socioeconomic deprivation, physical activity, alcohol frequency, years in full-time education, diabetes status, anxiety and depression, COPD, hypertension, inflammatory bowel disease, rheumatoid arthritis, obstructive sleep apnoea, and multiple sclerosis. For the fluid intelligence test, fully adjusted models additionally included years in full-time education. For the prospective memory test logistic regression was performed and the estimates reported are odds ratios. Minimally adjusted models for this test include age (years) and sex and fully adjusted models additionally adjusted for physical activity in the fully adjusted models. | | | | | | |

**Supplementary table 10: Association of common infections with cognitive decline, excluding follow up infections**

|  |  | **Minimally adjusted** | |  | **Fully adjusted model** | |
| --- | --- | --- | --- | --- | --- | --- |
|  | **No. of participants** | **β (95% CI)** | **P value** | **No. of participants** | **β (95% CI)** | **P value** |
| **Mean correct response time** (Difference in slope compared with no infection) | | | | | | |
| No infection | 10,336 | Reference | | 10,026 | Reference | |
| Any infection | 2,956 | 0.58 (0.0085 to 1.15) | 0.05 | 2,809 | 0.49 (-0.092 to 1.06) | 0.10 |
| **Visual memory** (Difference in slope compared with no infection) | | | | | | |
| No infection | 8,919 | Reference | | 8,638 | Reference | |
| Any infection | 2,562 | 0.00071 (-0.0031 to 0.0045) | 0.71 | 2,436 | 0.00028 (-0.0036 to 0.0041) | 0.89 |
| **Fluid intelligence** (Difference in slope compared with no infection) | | | | | | |
| No infection | 3,633 | Reference | | 3,622 | Reference | |
| Any infection | 1,070 | 0.0061 (-0.011 to 0.023) | 0.48 | 1,066 | 0.0064 (-0.011 to 0.024) | 0.47 |
|  | **No. of participants** | **OR (95% CI)** | **P value** | **No. of participants** | **OR (95% CI)** | **P value** |
| **Prospective memory** (Difference in slope compared with no infection) | | | | | | |
| No infection | 3,258 | Reference | | 3,188 | Reference | |
| Any infection | 926 | 0.74 (0.57 to 0.96) | 0.02 | 894 | 0.78 (0.60 to 1.02) | 0.07 |
| Linear Mixed models results with random intercept and random slope. For reaction time, visual memory (log transformed) and fluid intelligence tests, minimally adjusted: age(years), sex, time, baseline test score and time x infection status interaction term which represents the rate of decline by presence of infection with the difference in slope compared to that of no infection (reference group). For reaction time, fully adjusted models additionally adjusted for ethnicity, BMI, years in full-time education, physical activity, alcohol consumption, diabetes category, anxiety and depression, COPD, multiple sclerosis, hypertension and heart failure. For the visual memory test, fully adjusted models additionally adjusted for ethnicity, BMI, smoking status, socioeconomic deprivation, physical activity, alcohol frequency, years in full-time education, diabetes status, anxiety and depression, COPD, hypertension, inflammatory bowel disease, rheumatoid arthritis, obstructive sleep apnoea, and multiple sclerosis. For the fluid intelligence test, fully adjusted models additionally included years in full-time education. For the prospective memory test logistic regression was performed and the estimates reported are odds ratios. Minimally adjusted models for this test include age(years) and sex and fully adjusted models additionally adjusted for physical activity in the fully adjusted models. | | | | | | |

**Supplementary table 11: Association of common infections with hippocampal volume and white matter hyperintensities volume excluding follow up infections**

|  | **Minimally adjusted model** | | | **Fully adjusted model** | | |
| --- | --- | --- | --- | --- | --- | --- |
|  | **No. of participants** | **β Coefficient (95% Confidence Interval)** | **P Value** | **No. of participants** | **Β Coefficient**  **(95% Confidence Interval)** | **P Value** |
| **Total hippocampal volume (mm^3^)** | | | | | | |
| No infection | 12,275 |  |  | 11,911 |  |  |
| Any infection | 2,435 | -12.43 (-48.91 to 24.06) | 0.50 | 2,328 | 7.63 (-29.85 to 45.10) | 0.69 |
| **White matter hyperintensities (Exp B)** | | | | | | |
| No infection | 12,011 |  |  | 11,982 |  |  |
| Any infection | 2,386 | 1.05 (1.01 to 1.09) | 0.02 | 2,375 | 1.02 (0.98 to 1.06) | 0.35 |
| Estimates for any infection and site of infection. Fully adjusted models for hippocampal volume adjusted for age(years), sex, smoking, alcohol consumption, years in full-time education, diabetes category, chronic obstructive pulmonary disease, asthma, chronic kidney disease, chronic liver disease, hypertension, heart failure and psoriasis (n= 14,239). Fully adjusted models for log of volume of white matter hyperintensities included age(years), sex, BMI, anxiety and depression, chronic kidney disease, chronic liver disease, heart failure and psoriasis (n=14,357). | | | | | | |

**Supplementary table 12: Association of common infections with left and right hippocampal volume**

|  |  | **Minimally adjusted model** | |  | **Fully adjusted model** | |
| --- | --- | --- | --- | --- | --- | --- |
|  | **No. of participants** | **β Coefficient (95% Confidence Interval)** | **P Value** | **No. of participants** | **β**  **(95% Confidence Interval)** | **P Value** |
|  | | **Left hippocampal volume (mm^3^)** | | | | |
| No infection | 12,275 | Reference | | 11,911 | Reference | |
| Any infection | 2,435 | -8.00 (-28.02 to 12.01) | 0.43 | 2,328 | 3.76 (-16.80 to 24.32) | 0.72 |
| Other LRTI | 1,372 | -10.83 (-36.46 to 14.80) | 0.41 | 1,306 | 2.97 (-23.53 to 29.46) | 0.83 |
| UTI | 569 | -20.45 (-59.42 to 18.53) | 0.30 | 544 | -12.92 (-52.65 to 26.80) | 0.52 |
| SSTI | 431 | 16.15 (-27.94 to 60.24) | 0.47 | 417 | 26.28 (-18.44 to 71.00) | 0.25 |
|  | | **Right hippocampal volume (mm^3^)** | | | | |
| No infection | 12,275 | Reference | | 11,911 | Reference | |
| Any infection | 2,435 | -4.42 (-25.06 to 16.22) | 0.67 | 2,328 | 3.87 (17.38 to 25.11) | 0.72 |
| Other LRTI | 1,372 | 5.56 (-20.87 to 32.00) | 0.68 | 1,306 | 14.31 (-13.07 to 41.69) | 0.31 |
| UTI | 569 | -8.52 (-48.72 to 31.69) | 0.68 | 544 | 0.22 (-40.83 to 41.27) | 0.99 |
| SSTI | 431 | -22.05 (-67.53 to 23.43) | 0.34 | 417 | -16.32 (-62.54 to 29.90) | 0.49 |
| Estimates for any infection and site of infection. Fully adjusted models for left and right hippocampal volume adjusted for age(years), sex, smoking, alcohol consumption, years in full-time education, diabetes category, chronic obstructive pulmonary disease, asthma, chronic kidney disease, chronic liver disease, hypertension, heart failure and psoriasis (n= 14,239) | | | | | | |

**Supplementary Figure 1. Percentage of participants with and without infections stratified by age in the cognitive decline and neuroimaging cohort**


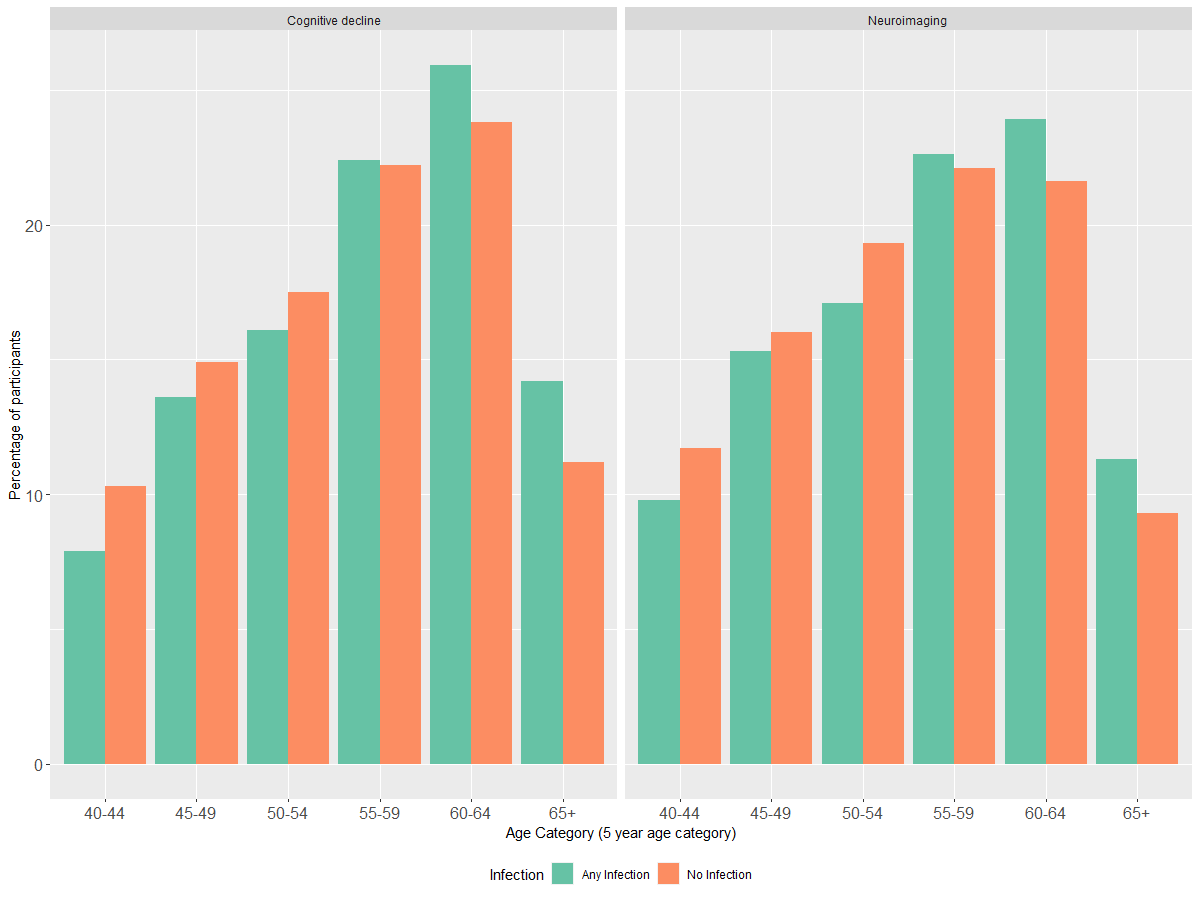


**Supplementary Figure 2. Association of common infections and cognitive decline, stratified by timing of common infections in the five years prior to baseline**

Linear Mixed models results with random intercept and random slope. For mean correct response time, visual memory (log transformed) and fluid intelligence tests, minimally adjusted: age(years), sex, time, baseline test score and time x infection status interaction term which represents the rate of cognitive decline by presence of infection with the difference in slope of infection compared to that of no infection (reference group). For mean correct response time, fully adjusted models additionally adjusted for ethnicity, BMI, years in full-time education, physical activity, alcohol consumption, diabetes category, anxiety and depression, COPD, multiple sclerosis, hypertension and heart failure. For the visual memory test, fully adjusted models additionally adjusted for ethnicity, BMI, smoking status, socioeconomic deprivation, physical activity, alcohol frequency, years in full-time education, diabetes status, anxiety and depression, COPD, hypertension, inflammatory bowel disease, rheumatoid arthritis, obstructive sleep apnoea, and multiple sclerosis. For the verbal-numerical reasoning test, fully adjusted models additionally included years in full-time education. For the prospective memory test logistic regression was performed and the estimates reported are odds ratios. Minimally adjusted models for this test include age(years) and sex and fully adjusted models additionally adjusted for physical activity in the fully adjusted models.

Linear Mixed models results with random intercept and random slope. For reaction time, visual memory and verbal-numeric reasoning tests. minimally adjusted: age(years), sex, time, baseline test score, interaction term with time x infection status allows the calculation of the rate of decline by presence of infection with no infection as the reference group. For reaction time, fully adjusted models additionally adjusted for ethnicity, BMI, years in full-time education, physical activity, alcohol consumption, diabetes category, anxiety and depression, COPD, multiple sclerosis, hypertension and heart failure. For the visual memory test, fully adjusted models additionally adjusted for ethnicity, BMI, smoking status, socioeconomic deprivation, physical activity, alcohol frequency, years in full-time education, diabetes status, anxiety and depression, COPD, hypertension, inflammatory bowel disease, rheumatoid arthritis, obstructive sleep apnoea, and multiple sclerosis. For the verbal-numerical reasoning test, fully adjusted models additionally included years in full-time education. For the prospective memory test logistic regression was performed and the estimates reported are odds ratios. Minimally adjusted models for this test include age(years) and sex and fully adjusted models additionally adjusted for physical activity in the fully adjusted models.

**REFERENCES**

1. Fry A, Littlejohns TJ, Sudlow C, et al. Comparison of Sociodemographic and Health-Related Characteristics of UK Biobank Participants With Those of the General Population. *American journal of epidemiology* 2017; **186**(9): 1026-34.

2. Sudlow C, Gallacher J, Allen N, et al. UK biobank: an open access resource for identifying the causes of a wide range of complex diseases of middle and old age. *PLoS medicine* 2015; **12**(3): e1001779-e.

3. Biobank U. UK Biobank. Primary care linked data version 1.0. 2019.

4. Lyall DM, Cullen B, Allerhand M, et al. Cognitive Test Scores in UK Biobank: Data Reduction in 480,416 Participants and Longitudinal Stability in 20,346 Participants. *PLOS ONE* 2016; **11**(4): e0154222.

5. Showcase UBD. Data-Field 20023. Mean time to correctly identify matches. 2021. <https://biobank.ctsu.ox.ac.uk/crystal/field.cgi?id=20023> (accessed 04/09/2021.

6. Biobank U. UK Biobank Brain Imaging Documentation version 1.8. 2020.

7. Littlejohns TJ, Holliday J, Gibson LM, et al. The UK Biobank imaging enhancement of 100,000 participants: rationale, data collection, management and future directions. *Nature communications* 2020; **11**(1): 2624.

8. Biobank U. UK Biobank. Repeat assessment: Participant Characteristics of responders vs. non-responders version 1.1. 2014.

9. Fawns-Ritchie C, Deary IJ. Reliability and validity of the UK Biobank cognitive tests. *PloS one* 2020; **15**(4): e0231627-e.

10. ISCED. International Standard Classification of Education 1997.

11. Schneider SL. The International Standard Classification of Education (ISCED-97): an evaluation of content and criterion validity for 15 European countries. 2008.

12. Townsend P, Phillimore P, Beattie A. Health and deprivation: inequality and the North: Routledge; 1988.

13. Showcase UBD. Data-Field 189. Townsend deprivation index at recruitment. 2021. <https://biobank.ctsu.ox.ac.uk/crystal/field.cgi?id=189#:~:text=Townsend%20deprivation%20index%20calculated%20immediately,which%20their%20postcode%20is%20located>. (accessed 04/09/2021.

14. Semmler A, Widmann CN, Okulla T, et al. Persistent cognitive impairment, hippocampal atrophy and EEG changes in sepsis survivors. *Journal of neurology, neurosurgery, and psychiatry* 2013; **84**(1): 62-9.

**UK Biobank Protocol**

**A1. Project Title**

The effect of common infections on cognitive decline, brain structure and incident dementia in the UK Biobank study

**A2. Research question and aim(s)**

Research question: Are common infections associated with cognitive decline, hippocampal volume, white matter hyperintensities and incident dementia?

Aims:

1. To investigate whether the presence of common infections is associated with worsening cognitive decline.
2. To investigate the association of common infections with hippocampal volume and white matter hyperintensities volume.
3. To investigate whether the presence of common infections is associated with incident dementia.

**A3. The background and scientific rationale of the proposed research project in general**

Dementia is a major public health burden. Currently, there are no effective treatments and as the global burden of dementia is forecast to rise rapidly, due to the ageing population, there is an urgent need to develop effective approaches to risk reduction. Recent evidence suggests that the age-specific incidence of dementia is declining in Europe and the USA, and this change has been attributed to changes in modifiable risk factors.[15-20] Thus, it is therefore important to identify other potentially preventable risk factors for dementia.

Infections have been proposed to play a role in the aetiology of dementia for decades. In our recently published systematic review of longitudinal studies, predominantly from Taiwan and the United States, individuals with common infections were at a greater risk of developing dementia compared to those without infections.[21] Common infections such as pneumonia, urinary tract infections and sepsis are well known to precipitate serious, reversible changes in cognition manifested as delirium. In turn, delirium and cognitive decline are major risk factors of dementia.[22-24] However, it remains unclear whether infections increase the risk of long-term changes in cognition. Findings from a US prospective study of older adults showed that individuals hospitalised with sepsis were associated with moderate to severe cognitive impairment.[25] However, other studies have found conflicting findings and face a number of important methodological limitations such as small sample sizes and inadequate adjustment for confounding. In addition, studies investigating the effect of type, frequency and timing of infections on multiple domains of cognitive impairment are scarce.

People with diabetes have impaired immune defence mechanisms and as a result they are at a greater risk of serious infections compared to people without diabetes, as evidenced by a large body of literature including a systematic review of 345 cohort and case control studies and a UK study of 102,493 individuals using primary care electronic health records.[26, 27] Diabetes is a well-known risk factor for cognitive decline and dementia.[28-30] In a recent systematic review and meta-analysis comprising 2.3 million individuals from 14 prospective studies, diabetes was associated with a 60% increased risk of all cause dementia and a 40% increased risk of non-vascular dementia.[31] Numerous neuropsychological studies have consistently found that people with diabetes perform worse on multiple domains of cognitive function tests, including speed processing and mental flexibility, compared to the general population.[32-34]

Diabetes has also been associated with neuropathological markers of cognitive dysfunction, such as hippocampal atrophy and white matter hyperintensities in neuroimaging studies.[35, 36] In turn, these structural brain measures are associated with cognitive decline and dementia. [37] Moreover, sepsis survivors and individuals with major infections have been linked with abnormalities in brain structure and lower brain volumes [38] [39] However, the association of infections on structural brain measures remains unclear and requires further study.

We aim to investigate the effect of the of common infections on cognitive decline using cognitive function tests and structural brain imaging data from the UK Biobank study linked to primary and secondary care records. In our secondary analyses, we will investigate the effect of type, frequency, clinical setting and timing of infections on cognitive decline. We will also investigate the association of common infections on cognitive decline in people with and without diabetes and the effect of infections on hippocampal volume, white matter hyperintensities and dementia.

**A4. The expected value of the research**

Infections and diabetes are potentially modifiable conditions with an increasing public health burden. Our study may provide a better understanding of the interrelationship between infections and diabetes with cognitive decline and the cognitive domains affected. Further, if infections and diabetes are associated with cognitive decline this may help inform intervention trials and public health strategies to reduce the risk of cognitive decline and dementia. Strategies could involve identifying populations most at risk of both infections and cognitive impairment, early recognition and treatment of infections, and approaches to increase vaccine uptake. Strategies among people with diabetes could include interventions to improve diabetes self-management and early identification of infections in people with diabetes.

A5. Please provide a lay summary of your research project in plain English, stating the aims, scientific rationale, project duration and public health impact:

As we age(years), changes occur in the brain that are expected as part of the normal aging process. This includes changes in memory, attention and how quickly the brain processes information. Cognitive decline occurs when these changes are beyond that expected of an individual based on their age and educational background. Having cognitive decline increases the risk of dementia, and cognitive decline often occurs years before dementia develops. Over the last few decades, increasing evidence suggests that preventable risk factors, such as education and heart disease, may increase the chances of developing cognitive decline and dementia. With this in mind, it is important to identify other potentially preventable risk factors. One such potentially modifiable risk factor could be infections.

Common infections, such as sepsis and urinary tract infections, often trigger reversible short-term changes in brain function. However, it is unclear whether these infections may also lead to long term changes in cognition. A number of studies suggest that individuals hospitalised with common infections are at an increased risk of developing cognitive changes that persist in the long term. However, evidence of this association remains unclear.

People with diabetes have a greater chance of developing cognitive decline compared to the general population. Studies have also shown they perform worse in tests that measure cognitive abilities. A decline in cognitive abilities may affect how individuals are able to self-manage their diabetes and may result in worsening of diabetes. People with diabetes are also known to have a higher risk of dementia.

Overall, we aim to investigate whether common infections (sepsis, lower respiratory tract-, urinary tract- and skin and soft tissue infections) are associated with differences in brain function, using data from the UK Biobank study. First, we will investigate whether the presence of common infections is associated with cognitive decline. Second, we will investigate whether common infections are associated with changes in brain structure. Third, we will investigate whether infections are associated with dementia.

Infections and diabetes are potentially modifiable and may therefore present a potential target to delay or prevent the onset of cognitive decline and dementia. Understanding how infections and diabetes work to affect brain function can help to develop effective strategies in reducing the burden of cognitive decline and dementia.

**A6. Study population**

We will include adults aged 40-69 years of age at recruitment for the UK Biobank study between 2006 and 2010. Specifically, we will only include individuals with linked primary care data (approximately 45% of UK Biobank cohort) and at least 12 months follow up in primary care records prior to baseline assessment. For our analyses on cognitive decline, we will only include individuals with valid measures of cognitive function completed at baseline and one or two follow-up visits (either the first repeat assessment date in 2012-13 and/or the imaging visit from 2014 onwards). We will exclude individuals with dementia and cognitive impairment at baseline. For our analyses focusing on infections and neuroimaging measures, we will only include individuals with baseline neuroimaging data.

**A7. Study design**

Historical cohort study using UK biobank data with linked primary and secondary care data

**5 years prior to baseline**

Measurements:

- Common infections identified in primary and secondary care records

**Baseline (2006-2010)**

Measurements:

- **Cognitive function tests**
- Reaction time (n=496,667)
- Pairs Matching (n=497,869)
- Fluid intelligence (n=165,456)
- Prospective memory (n=171,545)
- **Covariates** (sociodemographic, lifestyle variables and comorbidities)
- **Exclusions**

Prevalent dementia or cognitive impairment

**First repeat assessment (2012-2013)**

Measurements:

- **Cognitive function tests**
- Reaction time test (n=20,255)
- Pairs Matching (n=20,335)
- Fluid intelligence (n=20,111)
- Prospective memory (n=20,330)

**Imaging visit (2014+)**

Measurements:

- **Cognitive function tests**
- Reaction time test (n=45,620)
- Pairs Matching (n=45,930)
- Fluid intelligence (n=45,044)
- Prospective memory (n=45,906)
- **MRI brain imaging**
- Hippocampal volume (n=39,678)
- White matter hyperintensities (n=38,346)

Historical cohort study using UK biobank data with linked primary and secondary care data

**5 years prior to baseline**

Measurements:

- Common infections identified in primary and secondary care records

**Baseline (2006-2010)**

Measurements:

- **Cognitive function tests**
- Reaction time (n=496,667)
- Pairs Matching (n=497,869)
- Fluid intelligence (n=165,456)
- Prospective memory (n=171,545)
- **Covariates** (sociodemographic, lifestyle variables and comorbidities)
- **Exclusions**

Prevalent dementia or cognitive impairment

**First repeat assessment (2012-2013)**

Measurements:

- **Cognitive function tests**
- Reaction time test (n=20,255)
- Pairs Matching (n=20,335)
- Fluid intelligence (n=20,111)
- Prospective memory (n=20,330)

**Imaging visit (2014+)**

Measurements:

- **Cognitive function tests**
- Reaction time test (n=45,620)
- Pairs Matching (n=45,930)
- Fluid intelligence (n=45,044)
- Prospective memory (n=45,906)
- **MRI brain imaging**
- Hippocampal volume (n=39,678)
- White matter hyperintensities (n=38,346)

Fig 1. Study design and population

Source: <https://biobank.ndph.ox.ac.uk/showcase/> Accessed October 2020

**A8. Outcome**

**Cognitive decline**

Our primary outcome is cognitive decline. We will measure changes in cognitive function from baseline to follow up. We will include the following cognitive function tests which were all assessed at the baseline visit and follow up time points: reaction time, pairs matching, fluid intelligence and prospective memory.

*Reaction time*

The reaction time test was designed to assess speed processing and was measured using a computer version of the game ‘snap’. Participants were shown two cards on a touch screen and were instructed to press a button as quickly as possible when the symbols on the cards matched. We will assess the outcome measure for reaction time using the mean time (milliseconds) taken to correctly identify matches.

*Pairs matching*

The pairs matching test assessed for visual memory. For this test, participants were shown 6 pairs of cards with symbols for 5 seconds and were instructed to memorise the position of as many matching pairs of cards, in the fewest tries, as possible. The outcome measure will be the total number of incorrect matches in participants who completed the test.

*Fluid intelligence*

Fluid intelligence assessed verbal and numeric reasoning. For this test, participants were given two minutes to answer as many questions as possible. The questions required logic and reasoning ability. An example of a verbal reasoning question was “If Truda’s mother’s brother is Tim’s sister’s father, what relation is Truda to Tim?”. An example of a numeric reason question was: “If sixty is more than half of seventy-five, multiply twenty-three by three. If not subtract 15 from eighty-five.” Participants were given a number of possible responses to select from. The total number of correct answers to the 13 questions will be the outcome measure.

*Prospective memory*

The prospective memory test assessed participants’ ability to remember to perform an action in the future. Before participants completed the other tests, they were first instructed the following: “At the end of the games we will show you four coloured shapes and ask you to touch the Blue Square. However, to test your memory, we want you to actually touch the Orange Circle instead”. Participants were scored 1 for correct at first attempt and 0 for incorrect at first attempt.

Fluid intelligence and prospective memory tests were included when the baseline assessment tests had already been initiated, as a result the sample sizes for the tests at baseline is smaller than that of the reaction time and pairs matching tests.[40]

***Neuroimaging measures***

Structural brain MRI measures, white matter hyperintensities and hippocampal volume, were measured at the imaging visit from 2014 onwards. We will use T1 and T2 weighted FLAIR imaging technique measuring the total volume of white matter hyperintensities.

***Dementia***

Incident dementia will be defined using Read and ICD-10 codes from linked primary and secondary care records and mortality data. Dementia will be defined using a broad definition which will include Alzheimer’s disease and vascular dementia. Individuals who self-reported as having dementia at the baseline nurse interview and those with a history of dementia in their linked primary and secondary records will be excluded from the study. Our prevalent dementia codes will include administrative codes such as ‘dementia care plan’ as well as diagnostic codes.

**A9. Exposure**

Infection diagnosis 5 years prior to baseline

Repeat cognitive assessment (imaging visit)

Baseline assessment

2013

2010

Imaging visit

2006

Baseline

2014+

2012

First repeat cognitive function follow-up

Historical infections (5 years prior to baseline)

Person 1 ***(Exposed)***

Person 2 ***(Unexposed)***

Fig 2. Definition of exposure status

We will identify common infections using linked primary and secondary care records. These infections will comprise sepsis, pneumonia, other lower respiratory tract infections, urinary tract infections and skin and soft tissue infections. We will group all common infections into one category ‘any infection’ in order to determine the overall association of common infections with cognitive decline. Then we will group infections according to subtype of infection for our secondary analyses.

Infections will be identified in the 5 years prior to baseline. If individuals are diagnosed with more than one infection during this period, the earliest record of infection is taken. Participants diagnosed with infections during follow up will be classified as unexposed.

Individuals will be defined as having sepsis, lower respiratory tract infections or pneumonia if they have a clinical diagnostic code for these infections. Individuals will be diagnosed as having urinary tract infections or skin and soft tissue infections if they have both a clinical diagnostic code and a prescription for antibiotics on the same date.

**A10. Covariates**

Based on existing literature we will consider the following potential confounders. Information on demographic, lifestyle factors and comorbidities will be identified using data from the baseline assessment questionnaires and linked primary and secondary care data. Demographic variables will include age(years), sex, ethnicity (white, south Asian, black, mixed or other), education and socioeconomic background which will be obtained from the initial interview assessment at baseline. Socioeconomic status will be measured using the Townsend Deprivation Index, based on postal codes, and measured using quintiles ranging from least deprived to most deprived. Potential lifestyle factors will include body mass index, smoking (never smoker, former smoker or current smoker), alcohol intake frequency (daily or almost daily, three or four times a week, once or twice a week one to three times a month, special occasions only, never) and physical activity (number of days spent doing moderate or physical activity). Diabetes status will be ascertained using HbA1c and medication history at baseline. Other comorbidities include hypertension, stroke, myocardial infarction, chronic kidney disease, chronic liver disease, traumatic brain injury, asthma, chronic obstructive pulmonary disease, severe mental illness, depression, anxiety, inflammatory autoimmune conditions, psychiatric comorbidity and inflammatory disease, and medication use. APOE status will be ascertained using genetic data.

**A11. Statistical analyses**

First, we will perform descriptive analyses to describe the characteristics (sociodemographic, lifestyle factors and comorbidities) of participants excluded from the study from recruitment to repeat assessments, using numbers and percentages for categorical data and mean and median values and interquartile range for continuous data. We will also perform further descriptive analyses to investigate the characteristics of participants diagnosed with infections and dementia. Second, we will describe the age-specific mean cognitive function scores at baseline and follow up assessment in participants with and without common infections. We will stratify age into the following age groups: 40-44, 45-49, 50-54, 55-59, 60-64, 65+.

Third, for cognitive function tests with continuous outcomes (reaction time, pairs matching and fluid intelligence), we will use linear mixed models with random intercept and random slope. For binary outcome measures (prospective memory), we will use multiple logistic regression models in individuals with correct recall at baseline, and we will adjust for time elapsed since baseline measurement. To select the confounders appropriate for inclusion, we will use a directed acyclic graph. We will then use a backwards deletion approach to identify screen potential confounders. Fourth, we will investigate the association between type (sepsis, pneumonia, other lower respiratory tract infections, urinary tract infections and skin and soft tissue infections), frequency, clinical setting (GP vs Hospital recorded infections) and timing (time since infection diagnosis) of infections. We will explore whether the effect of infections on cognition differs by glycaemic status and test the presence of effect modification by fitting an interaction term.

Fifth, we will perform cross-sectional analyses, based on the time point of the imaging visit, using multiple linear regression models to estimate the association between common infections and each structural brain MRI marker (hippocampal volume and white matter hyperintensities).

Lastly, we will use Cox regression models to estimate the association between common infections and incident dementia. We will test for the Cox proportional hazards assumption using log-log plots and Schoenfeld residuals.

We will consider the following sensitivity and additional secondary analyses in which we will:

1. Repeat main analyses excluding individuals with less than 5 years of follow up prior to baseline assessment given that infections will be captured in the 5 years prior to baseline
2. Repeat our main analyses excluding participants diagnosed with infections during follow up
3. Explore effect modification of the association between common infections and cognitive decline by Apolipoprotein (APOE) genotype
4. Stratify by sex to compare the effect of infections on cognitive decline in men and women.
5. Stratify by dementia subtype in order to explore the incidence of dementia according to subtypes of dementia (Alzheimer’s disease and vascular dementia).
6. Investigate the longitudinal association of common infections with hippocampal volume and white matter hyperintensities

**A12. Plans for confounding**

Our final model will use a parsimonious model approach to include confounders specified in section A9.

**A13. Missing data**

We expect missing data on all four cognitive function tests and covariates such as ethnicity, BMI and education. We will describe the pattern of our missing data (whether our data is missing completely at random, missing at random or missing not at random) and choose an appropriate method for dealing with the missing data.

**A14. Feasibility counts**

- Approximately 181,631 participants aged 40 years and older in the UK Biobank study with linked primary and secondary care data with at least 12 months follow up prior to baseline assessment and no history of dementia or cognitive impairment. Of these participants, 161,490 had at least 5 years of follow up prior to baseline assessment.
- From our preliminary analysis, we found that 17,127 participants had at least one follow-up measurement for the pairs matching test, 17,040 individuals for the reaction time test and 5,934 for the fluid intelligence test. 5,256 participants had a correct answer for the prospective memory test and at least one follow up measurement.
- In our preliminary analysis of GP recorded infections, 3,817 participants were diagnosed with any infection, 2,903 had lower respiratory tract infections (pneumonia =34, other lower respiratory tract infections =2,869), 424 had urinary tract infections, 371 has skin and soft tissue infections and 119 participants had sepsis.

**A15. Sample size calculation**

Based on our feasibility counts described above, we estimated the number of participants with follow cognitive function data and individuals with and without any infection (A.14). Using data from a previous UK Biobank study, the overall raw mean score of the cognitive function tests were as follows: 6.98 (sd 2.09) for fluid intelligence 4.90 (sd 3.11) for pairs matching and 552.23 (sd 212.01) for reaction time.[40] We estimate that at 90% power and 5% significance level, a minimum detectable difference of 0.2, 0.19 and 12.65 in mean score will be detected between individuals with and without infections for the fluid intelligence, pairs matching and reaction time tests, respectively.

**A16. Strengths and limitations**

Strengths of this study include the large size of the UK biobank study population, multiple measures of cognitive function, assessment of cognitive function at multiple timepoints, extensive data on many covariates and the linkage to primary and secondary care records.

However, there are a number of limitations. First, as individuals were recruited into the UK Biobank study aged 40-69, our findings may not be generalisable to older adults. However, trajectories of cognitive decline are recognised to be underway years before onset of dementia thus a better understanding of the timing of cognitive decline following infection could inform dementia risk reduction strategies.[22] Second, loss of follow up is an issue in the UK Biobank cohort, particularly regarding cognitive measures. There is potential for bias if participants with more severe infections or poorer cognitive ability may be more likely to be lost at follow up for cognitive function tests and imaging visits [41] Further, only participants who had an email address were able to take the cognition tests at follow up, which may mean that the characteristics of those at baseline and follow up may differ. Moreover, there are differences in the way in which cognitive function tests were carried out at baseline and at follow up. At baseline, all cognition tests were performed using a touch screen interface, whereas at follow up, a mouse interface was used. This may contribute to variability in cognitive performance over time. Fourth, cognitive function tests were brief, non-standardised and lacked external validity.[40] However, a recent prospective study using UK Biobank data demonstrated an association between the baseline cognitive function tests and incident dementia, validating their use in dementia-related research.[42] Finally, participants in the UK Biobank are generally healthier than the general population. However, this is not a limitation when investigating exposure and outcomes associations and the findings may still be widely generalisable.[1]

**ReferencesREFERENCES**

[1] Fry A, Littlejohns TJ, Sudlow C, Doherty N, Adamska L, Sprosen T, et al. Comparison of Sociodemographic and Health-Related Characteristics of UK Biobank Participants With Those of the General Population. American journal of epidemiology. 2017;186:1026-34.

[2] Sudlow C, Gallacher J, Allen N, Beral V, Burton P, Danesh J, et al. UK biobank: an open access resource for identifying the causes of a wide range of complex diseases of middle and old age. PLoS medicine. 2015;12:e1001779-e.

[3] Biobank U. UK Biobank. Primary care linked data version 1.0. 2019.

[4] Lyall DM, Cullen B, Allerhand M, Smith DJ, Mackay D, Evans J, et al. Cognitive Test Scores in UK Biobank: Data Reduction in 480,416 Participants and Longitudinal Stability in 20,346 Participants. PLOS ONE. 2016;11:e0154222.

[5] Showcase UBD.Data-Field 20023. Mean time to correctly identify matches.2021.<https://biobank.ctsu.ox.ac.uk/crystal/field.cgi?id=20023>. Accessed: 04/09/2021

[6] Ratcliff R. Methods for dealing with reaction time outliers. Psychol Bull. 1993;114:510-32.

[7] Biobank U. UK Biobank Brain Imaging Documentation version 1.8. 2020.

[8] Littlejohns TJ, Holliday J, Gibson LM, Garratt S, Oesingmann N, Alfaro-Almagro F, et al. The UK Biobank imaging enhancement of 100,000 participants: rationale, data collection, management and future directions. Nature communications. 2020;11:2624.

[9] Biobank U. UK Biobank. Repeat assessment: Participant Characteristics of responders vs. non-responders version 1.1. 2014.

[10] Fawns-Ritchie C, Deary IJ. Reliability and validity of the UK Biobank cognitive tests. PloS one. 2020;15:e0231627-e.

[11] ISCED. International Standard Classification of Education 1997.

[12] Schneider SL. The International Standard Classification of Education (ISCED-97): an evaluation of content and criterion validity for 15 European countries. 2008.

[13] Townsend P, Phillimore P, Beattie A. Health and deprivation: inequality and the North: Routledge; 1988.

[14] Showcase UBD.Data-Field 189. Townsend deprivation index at recruitment.2021.<https://biobank.ctsu.ox.ac.uk/crystal/field.cgi?id=189#:~:text=Townsend%20deprivation%20index%20calculated%20immediately,which%20their%20postcode%20is%20located>. Accessed: 04/09/2021

[15] Matthews FE, Stephan BC, Robinson L, Jagger C, Barnes LE, Arthur A, et al. A two decade dementia incidence comparison from the Cognitive Function and Ageing Studies I and II. Nature communications. 2016;7:11398.

[16] van Bussel EF, Richard E, Arts DL, Nooyens ACJ, Coloma PM, de Waal MWM, et al. Dementia incidence trend over 1992-2014 in the Netherlands: Analysis of primary care data. PLoS medicine. 2017;14:e1002235-e.

[17] Grasset L, Brayne C, Joly P, Jacqmin-Gadda H, Peres K, Foubert-Samier A, et al. Trends in dementia incidence: Evolution over a 10-year period in France. Alzheimer's & dementia : the journal of the Alzheimer's Association. 2016;12:272-80.

[18] Qiu C, von Strauss E, Backman L, Winblad B, Fratiglioni L. Twenty-year changes in dementia occurrence suggest decreasing incidence in central Stockholm, Sweden. Neurology. 2013;80:1888-94.

[19] Satizabal CL, Beiser AS, Chouraki V, Chêne G, Dufouil C, Seshadri S. Incidence of Dementia over Three Decades in the Framingham Heart Study. The New England journal of medicine. 2016;374:523-32.

[20] Rocca WA, Petersen RC, Knopman DS, Hebert LE, Evans DA, Hall KS, et al. Trends in the incidence and prevalence of Alzheimer's disease, dementia, and cognitive impairment in the United States. Alzheimer's & dementia : the journal of the Alzheimer's Association. 2011;7:80-93.

[21] Muzambi R, Bhaskaran K, Brayne C, Davidson JA, Smeeth L, Warren-Gash C. Common Bacterial Infections and Risk of Dementia or Cognitive Decline: A Systematic Review. Journal of Alzheimer's Disease. 2020;76:1609-26.

[22] Amieva H, Jacqmin-Gadda H, Orgogozo JM, Le Carret N, Helmer C, Letenneur L, et al. The 9 year cognitive decline before dementia of the Alzheimer type: a prospective population-based study. Brain : a journal of neurology. 2005;128:1093-101.

[23] Davis DH, Muniz Terrera G, Keage H, Rahkonen T, Oinas M, Matthews FE, et al. Delirium is a strong risk factor for dementia in the oldest-old: a population-based cohort study. Brain : a journal of neurology. 2012;135:2809-16.

[24] Davis DJ, Muniz-Terrera G, Keage HD, et al. Association of delirium with cognitive decline in late life: A neuropathologic study of 3 population-based cohort studies. JAMA Psychiatry. 2017;74:244-51.

[25] Iwashyna TJ, Ely EW, Smith DM, Langa KM. Long-term cognitive impairment and functional disability among survivors of severe sepsis. Jama. 2010;304:1787-94.

[26] Abu-Ashour W, Twells L, Valcour J, Randell A, Donnan J, Howse P, et al. The association between diabetes mellitus and incident infections: a systematic review and meta-analysis of observational studies. BMJ Open Diabetes Research &amp;amp; Care. 2017;5.

[27] Carey IM, Critchley JA, DeWilde S, Harris T, Hosking FJ, Cook DG. Risk of Infection in Type 1 and Type 2 Diabetes Compared With the General Population: A Matched Cohort Study. Diabetes care. 2018;41:513-21.

[28] Biessels GJ, Despa F. Cognitive decline and dementia in diabetes mellitus: mechanisms and clinical implications. Nature reviews Endocrinology. 2018;14:591-604.

[29] Biessels GJ, Staekenborg S, Brunner E, Brayne C, Scheltens P. Risk of dementia in diabetes mellitus: a systematic review. The Lancet Neurology. 2006;5:64-74.

[30] Cheng G, Huang C, Deng H, Wang H. Diabetes as a risk factor for dementia and mild cognitive impairment: a meta-analysis of longitudinal studies. Internal medicine journal. 2012;42:484-91.

[31] Chatterjee S, Peters SA, Woodward M, Mejia Arango S, Batty GD, Beckett N, et al. Type 2 Diabetes as a Risk Factor for Dementia in Women Compared With Men: A Pooled Analysis of 2.3 Million People Comprising More Than 100,000 Cases of Dementia. Diabetes care. 2016;39:300-7.

[32] Brands AM, Biessels GJ, de Haan EH, Kappelle LJ, Kessels RP. The effects of type 1 diabetes on cognitive performance: a meta-analysis. Diabetes Care. 2005;28:726-35.

[33] van den Berg E, Kloppenborg RP, Kessels RPC, Kappelle LJ, Biessels GJ. Type 2 diabetes mellitus, hypertension, dyslipidemia and obesity: A systematic comparison of their impact on cognition. Biochimica et Biophysica Acta (BBA) - Molecular Basis of Disease. 2009;1792:470-81.

[34] Palta P, Schneider ALC, Biessels GJ, Touradji P, Hill-Briggs F. Magnitude of cognitive dysfunction in adults with type 2 diabetes: a meta-analysis of six cognitive domains and the most frequently reported neuropsychological tests within domains. J Int Neuropsychol Soc. 2014;20:278-91.

[35] Hayashi K, Kurioka S, Yamaguchi T, Morita M, Kanazawa I, Takase H, et al. Association of cognitive dysfunction with hippocampal atrophy in elderly Japanese people with type 2 diabetes. Diabetes research and clinical practice. 2011;94:180-5.

[36] Gold SM, Dziobek I, Sweat V, Tirsi A, Rogers K, Bruehl H, et al. Hippocampal damage and memory impairments as possible early brain complications of type 2 diabetes. Diabetologia. 2007;50:711-9.

[37] Silbert LC, Nelson C, Howieson DB, Moore MM, Kaye JA. Impact of white matter hyperintensity volume progression on rate of cognitive and motor decline. Neurology. 2008;71:108-13.

[38] Semmler A, Widmann CN, Okulla T, Urbach H, Kaiser M, Widman G, et al. Persistent cognitive impairment, hippocampal atrophy and EEG changes in sepsis survivors. Journal of Neurology, Neurosurgery &amp;amp; Psychiatry. 2013;84:62.

[39] Walker KA, Gottesman RF, Wu A, Knopman DS, Mosley Jr. TH, Alonso A, et al. Association of Hospitalization, Critical Illness, and Infection with Brain Structure in Older Adults. Journal of the American Geriatrics Society. 2018;66:1919-26.

[40] Lyall DM, Cullen B, Allerhand M, Smith DJ, Mackay D, Evans J, et al. Cognitive Test Scores in UK Biobank: Data Reduction in 480,416 Participants and Longitudinal Stability in 20,346 Participants. PloS one. 2016;11:e0154222-e.

[41] Matthews FE, Chatfield M, Freeman C, McCracken C, Brayne C, Cfas MRC. Attrition and bias in the MRC cognitive function and ageing study: an epidemiological investigation. BMC Public Health. 2004;4:12.

[42] Calvin CM, Wilkinson T, Starr JM, Sudlow C, Hagenaars SP, Harris SE, et al. Predicting incident dementia 3-8 years after brief cognitive tests in the UK Biobank prospective study of 500,000 people. Alzheimer's & dementia : the journal of the Alzheimer's Association. 2019;15:1546-57.

[43] Muzambi R. The effect of common infections on cognition and dementia in people with and without diabetes. London London School of Hygiene and Tropical Medicine; 2022.

**Changes to protocol**

1. This protocol was developed as part of a PhD thesis. Additional analyses on dementia were developed specifically to be included in the thesis while we aimed to publish the remaining analyses in a journal.[43]
2. We did not carry out aim c investigating the longitudinal association of infections and neuroimaging measures due to the paucity of data released for the first repeat imaging visit.
3. We were unable to explore potential effect modification by APOE4 genotype (secondary analysis aim 3) due to issues regarding downloading and extracting genetic data on London School of Hygiene and Tropical Medicine servers.
